# Ultra-high field cardiac MRI in large animals and humans for translational cardiovascular research

**DOI:** 10.3389/fcvm.2023.1068390

**Published:** 2023-05-15

**Authors:** Laura M. Schreiber, David Lohr, Steffen Baltes, Ulrich Vogel, Ibrahim A. Elabyad, Maya Bille, Theresa Reiter, Aleksander Kosmala, Tobias Gassenmaier, Maria R. Stefanescu, Alena Kollmann, Julia Aures, Florian Schnitter, Mihaela Pali, Yuichiro Ueda, Tatiana Williams, Martin Christa, Ulrich Hofmann, Wolfgang Bauer, Brenda Gerull, Alma Zernecke, Süleyman Ergün, Maxim Terekhov

**Affiliations:** ^1^ Department of Cardiovascular Imaging and Chair of Molecular and Cellular Imaging, Comprehensive Heart Failure Center Wuerzburg (CHFC), University Hospital Wuerzburg, Wuerzburg, Germany; ^2^ Institute for Hygiene and Microbiology, University of Wuerzburg, Wuerzburg, Germany; ^3^ Department of Internal Medicine I/Cardiology, University Hospital Wuerzburg, Wuerzburg, Germany; ^4^ Department of Radiology, University Hospital Wuerzburg, Wuerzburg, Germany; ^5^ Institute of Anatomy and Cell Biology, Julius-Maximilians-University, Wuerzburg, Germany; ^6^ Department of Cardiovascular Genetics, Comprehensive Heart Failure Center Wuerzburg, University Hospital Wuerzburg, Wuerzburg, Germany; ^7^ Institute of Experimental Biomedicine, University Hospital Wuerzburg, Wuerzburg, Germany

**Keywords:** ultrahigh-field MRI, large animal models, translational research, research infrastructure, heart, organoid, pig, cardiovascular MRI

## Abstract

A key step in translational cardiovascular research is the use of large animal models to better understand normal and abnormal physiology, to test drugs or interventions, or to perform studies which would be considered unethical in human subjects. Ultrahigh field magnetic resonance imaging (UHF-MRI) at 7 T field strength is becoming increasingly available for imaging of the heart and, when compared to clinically established field strengths, promises better image quality and image information content, more precise functional analysis, potentially new image contrasts, and as all *in-vivo* imaging techniques, a reduction of the number of animals per study because of the possibility to scan every animal repeatedly. We present here a solution to the dual use problem of whole-body UHF-MRI systems, which are typically installed in clinical environments, to both UHF-MRI in large animals and humans. Moreover, we provide evidence that in such a research infrastructure UHF-MRI, and ideally combined with a standard small-bore UHF-MRI system, can contribute to a variety of spatial scales in translational cardiovascular research: from cardiac organoids, Zebra fish and rodent hearts to large animal models such as pigs and humans. We present pilot data from serial CINE, late gadolinium enhancement, and susceptibility weighted UHF-MRI in a myocardial infarction model over eight weeks. In 14 pigs which were delivered from a breeding facility in a national SARS-CoV-2 hotspot, we found no infection in the incoming pigs. Human scanning using CINE and phase contrast flow measurements provided good image quality of the left and right ventricle. Agreement of functional analysis between CINE and phase contrast MRI was excellent. MRI in arrested hearts or excised vascular tissue for MRI-based histologic imaging, structural imaging of myofiber and vascular smooth muscle cell architecture using high-resolution diffusion tensor imaging, and UHF-MRI for monitoring free radicals as a surrogate for MRI of reactive oxygen species in studies of oxidative stress are demonstrated. We conclude that UHF-MRI has the potential to become an important precision imaging modality in translational cardiovascular research.

## 1. Introduction

Magnetic Resonance Imaging (MRI) is of high relevance in clinical decision making, and increasingly in cardiovascular research. It allows to non-invasively visualize morphology and measure a wide variety of parameters highly relevant in the research process such as cardiac function, myocardial viability, metabolism, tissue microstructure, or myocardial inflammation. Preclinical studies in large animal models are key in the translation from basic research to the human application e.g., in drug and contrast agent development, development of interventional or surgical procedures, tissue engineering applications, or safety studies on medical devices. Furthermore, studies of physiology and pathophysiology of the cardiovascular system in large animal models have a long history (1). Many countries (2, 3) aim to completely replace them long-term (4), but still they are of high relevance to provide unique information which cannot be obtained from replacement methods such as cell cultures and organoids (5, 6), computer simulations (7) or combinations thereof (8). Moreover, they allow testing methodologies which would be considered unsafe in humans such as application of untested drugs or contrast agents (9), or medical devices (10). Other applications are studies which would be unethical in humans, e.g., the use of open chest models in research (11) or resuscitation research (12, 13). Another application addresses experiments impractical in humans, e.g., very long-duration scans to obtain very high spatial or parametric information not available in normal clinical scans.

From an infrastructural and a technical point of view, MRI in large animals such as the pig, sheep, or dog is usually performed under suboptimal conditions. Commercial large animal MRI systems are not available, and whole-body UHF-MRI systems are expensive often preventing installation in translational research institutions. Thus, UHF-MRI in translational research is typically performed on whole-body MRI systems developed for clinical use and in environments dedicated to scanning human subjects. Radiofrequency (RF) coils are developed with the anatomy and shape of human subjects in mind, which often are very different from those of large animals (14, 15). MRI techniques such as pulse sequences and measurement protocols are provided with clinical demands in mind such as rapid scanning to reduce stress for the ill patient, and/or to increase patient throughput. Safety limitations regarding RF-induced tissue heating (specific absorption rate, SAR) or the risk of induction of peripheral nerve stimulation by rapid switching of gradient fields are in effect with the consequence of further limiting measurements compared to what may be feasible or desirable in research from a physics, technical, or experimental perspective.

Dual use of MRI systems in humans and animals requires particular attention to minimize hygienical risks such as zoonotic transmission and may also be limited by further ethical and psychological considerations such as the need to prevent direct contact between humans and animals.

Research is particularly demanding with regard to the permanent quest for the best data quality achievable that may well go beyond what is needed in diagnostic imaging for clinical decision making. Furthermore, some well-established animal models such as the Göttingen (16) or Aachen (17) minipigs are smaller than the normal adult human subject and, thus, need smaller voxels to achieve adequate spatial resolution in relation to subject size. Considering this, there is a particular need for the use of an imaging methodology that promises the highest possible signal-to-noise ratios (SNR) that is feasible in all subjects used during experimentation. Theoretically, keeping all other imaging parameters similar, the MRI signal *s* is

(1) $$s\propto B_{0\;}^{\gamma }\times B_{1}^{-}(\vec{r})$$

where *B*_0_ denotes the static field strength of the MRI system (7 T), and $B_{1}^{-}$ the sensitivity of the receive coil at position $\vec{r}$ (18). Experimental reports on the exponent *γ* of *B*_0_ are on the order of 1.65 for the brain (19). For the heart, an increase of the SNR was reported to be up to a factor of 2.1 (20) and 1.65 (21) when compared with 1.5 T and 3 T field strength, respectively.

Whatever the exact value of *γ* is, equation (1) demonstrates two key components of our UHF-MRI concept: Increasing the field strength has a supralinear effect on the MRI signal, and $B_{1}^{-}$ needs to be maximized i.e., RF coils with body size and body shape-adapted geometry are essential to minimize the geometrical distance between the cardiac tissue and the RF coil. An increased MRI signal *s* allows for an increase of the SNR, in spatial resolution, and/or a decrease of scan time per image.

Cardiac 7 T MRI in humans, however, is still not widely available because of methodological and technological challenges at high fields which are still an active area of research: (i) *B*_0_ (22) and $B_{1}^{+}$ (i.e., of the high-frequency transmit $B_{1}^{}$ field) inhomogeneities, (ii) excessive tissue heating, (iii) limited availability of optimized commercial pulse sequences (23), (iv) limited availability of commercial RF coil concepts for body (24) and cardiac (25, 26) imaging, (v) parallel transmit technology (27), (vi) unclear and not well-established safety concepts (28), (vii) and detrimental effects of the high field strength on the electrocardiogram (29–33) which may result in suboptimal synchronization of imaging with cardiac motion.

Installation of an MRI system is usually organized and executed by the vendor of the MRI system in collaboration with the administrative and technical staff of the hospital or research organization. However, demands of a dedicated infrastructure aiming for dual use of an MRI installation in large animals and humans are well beyond standard procedures and require new concepts: the mentioned ethical and hygiene aspects introduce additional demands: dedicated equipment is needed which may not be available commercially [e.g., devices compatible with a field strength higher than that of conventional clinical MRI systems (≦3 T)], standard hygiene measures may not be compatible with substance compatibility lists of the MRI system's vendor, the use of inert or explosive gases for MRI measurements may be requested from the researchers, or advanced electrical and physiological monitoring may be needed to safeguard the subject's wellbeing.

Therefore, we here present an UHF-MRI infrastructure for translational cardiovascular research in pigs and humans. We describe the dual use concept and implementation. We only focus on non-standard demands of MRI system and facility installation which go well beyond those of normal clinical and preclinical UHF-MRI system installations typically used at cardiovascular imaging sites. Moreover, we propose a RF coil concept for achieving optimal SNR in serial measurements in pigs, and we present proof-of-principle data demonstrating the feasibility of clinical and experimental cardiovascular-research directed imaging across scales, from cardiac organoids and cardiovascular tissue specimens, mice, pigs to humans.

## 2. Materials and methods

### 2.1. General UHF-MRI concept

UHF-MRI installation was performed at the Comprehensive Heart Failure Center (CHFC) in Wuerzburg (Germany) which is a research center combining basic and applied research with clinical research in patients with heart failure and its comorbidities. The long-term vision is the prevention of heart failure. As part of this research, imaging and imaging research are one of the cornerstone activities of the CHFC. Research activities are aiming at a fruitful interplay between basic research and translation including the development of cutting-edge UHF-MRI methodology as well as clinical and preclinical development of new treatments of heart failure, including new drugs.

The 7 T MRI installation was performed in a new research building. It comprises of two 7 T MRI systems, a latest technology whole-body 7 T MRI system (Siemens Magnetom Terra, Siemens Healthineers, Erlangen, Germany) for large animal (mainly pigs) and human scanning, and a Bruker Pharmascan 70/16 small-animal MRI system for rodent and experimental imaging. Equal field strength of both scanners allows for translation of results of MR properties of tissues and contrast agents from one system to the other, while the different object sizes necessitate different peak gradient strength and slew rates.

The whole-body UHF-MRI system is the main scope of this manuscript since it is a new instrument to translational cardiovascular research, and since the whole research infrastructure was built around it to guarantee optimal research conditions for now, and also to be flexible for the future for yet unknown but certainly developing new demands of the users. This MRI system is equipped with a passively and actively shielded 270 cm length zero boil-off 7 T magnet (Magnetom Terra, Siemens Healthineers, Erlangen, Germany), XR-gradients comprising of the SR gradient coil with 60 cm bore, and 80 mT/m and 200 mT/m/ms peak gradient strength and slew rate, respectively. The gradient system is specified for 100% duty-cycle, peak effective gradient strength and slew rate of 139 mT/m and 346 mT/m/ms, respectively. Moreover, the system includes a 3rd-order shim system correcting Z3, Z2X, Z2Y, and Z(X2-Y2) terms, whereas X3, Y3, and XYZ are not implemented. An 8.4 kW RF power amplifier is used in connection with a single channel transmission system. In a dedicated “research mode” eight 2 kW TX amplifiers can be used for *B*_1_-shimming or parallel transmission. The digital receive systems allow for signal reception of 32 channels at 10 Msamples/channel/sec using 32-bit data. The receive system's noise figure is ≤0.9. As currently no FDA or CE certification is available for cardiac MRI (34), the MRI system was installed without any certification. An upgrade of the RF-system for imaging and spectroscopy of other nuclei than hydrogen has been installed recently.

For large animal and human imaging, two modes of synchronization of the measurement with the heartbeat are available: the scanner-integrated wireless three-electrode electrocardiogram-triggering option, and an acoustic triggering device (EasyACT, MRI Tools, Berlin, Germany) (29, 35).

The Bruker Pharmascan 70/16 (Bruker BioSpin MRI GmbH, Ettlingen, Germany) 7 T small animal MRI system has 90 mm inner diameter gradients with 760 mT/m gradient strength and 6,840 mT/m/ms slew rate. It includes a cryogenically cooled ^1^H coil for optimized cardiac imaging and complements the MRI infrastructure for rodent, cardiac organoid imaging, and high-resolution *ex-vivo* imaging in excised heart specimens, or phantoms.

### 2.2. Concept for dual use in large animals and human

Dual use of a whole-body MRI system in both humans and large animals needed special attention (c.f., Tables 1, 2) which has been worked out in close collaboration with the hospital's Infection Control Unit (UV). Moreover, during the planning stage and the construction phase of the research building the dual use concept was discussed with both the Government of the District of Lower Franconia and the Veterinary Office of Wuerzburg before job-site inspection and before the official declaration of approval of the overall infrastructure.

**Table 1:** Special issues arising in and from translational MRI research.

Issue	Problem
Scanning of both humans and animals in the same MR scanner	risk of zoonosis ethical considerations smell nuisance irritation of patients, volunteers, and staff in and outside the facility by sighting animals
Potentially very long measurement times for scans (on the order of hours) and low SNR measurements	Image or spectral artifacts from RF interference induced by TV and radio stations, building equipment (e.g. main air-condition system), electric power stations, and supplies, other RF emitting sources (e.g., neon lamps, computers, and periphery, or electron microscopes)
Use of potentially dangerous substances	Risk of combustion or explosion by sparking within MRI system, RF coils, or facility infrastructure, in case of use of flammable liquids or gases (e.g., hydrogen) Risk of asphyxia (if oxygen displacing heavy gases are used, e.g. perfluorinated gases)
Use of MR-incompatible devices, or devices attracted by the magnetic field	Destruction or malfunction of the device Magnetoattraction, injury in staff or humans near MRI scanner or inside the MRI system's bore

**Table 2:** Issues and provisions taken for the translational research MRI system.

Issue	Provisions taken
Interference between Man and Animal	
Risk of zoonosis	Separate equipment for human and animal use (separate scanner tables and MRI coils, storage of consumables in different places) Standard operating procedures (SOPs) for operation and disinfection (e.g., no human measurement after animal experiments before disinfection of scanner room and animal preparation room) Access restriction to the facility for immunocompromised patients; no studies in these patients. Use of pathogen-free animals (optional)
Ethical considerations related to the simultaneous use of the MRI system	Arrangement of access routes and facilities to prevent simultaneous presence of humans and animals in the same space Organizational concept (no animal experiment per day before last human scanned) Approval of human studies within the facility by the ethics committee
Smell nuisance by animals	High-power air condition and ventilation (12 times/h) Excess pressure within scanner room to facilitate rapid removal Air-locks between the facility and hospital hallways to prevent smell leaving the area
Irritation of patients and staff by the sighting of animals	Separate access ways into and out of the facility for humans and animals
Special requirements in research MRI	
Reduced RF interfer­ence for MRI application with very low SNR and very long measurement times on the order of hours	Faraday cage with extra-thick walls shielding below and above the cage to prevent interference from building equipment and appliances (air condition, electric power stations, and supplies, …) e.g., from the building's main air condition system on the floor below MRI facility, and other sources like electric power stations and supplies e.g., the departments radiologic digital archive. Control of shielding efforts by RF shielding measurement.
Excessive noise due to long mea­sure­ments with strong gradients (within MRI facility and outside)	Additional noise dampening by sound insulating wool between the building's concrete structure and Faraday cage.
Use of non-MR-compatible and magnetic devices within scanner room	Bolts in walls and floor for fixation of devices with straps to prevent magneto-mechanical attraction
Use of special gases	
Use of oxygen displacing gases (e.g., perfluorinated gases like SF_6_ and C_2_F_6_)	Gas warning system to give acoustic and optical warning in case of excessive gas concentration Storage of gas bottles in dedicated and ventilated gas containers
The danger of explosion by use of hydrogen and parahydrogen	Explosive prevention and protection zone in scanner and animal preparation room (no ignition sources, illumination by optical fibers) Delivery of hydrogen gas from animal preparation room through metal pipes and sealed plastic pipes Explosion-certified air condition and ventilation technology in the animal preparation room. Storage of hydrogen bottles in dedicated and ventilated gas lockers Gas warning system to give acoustic and optical warning in case of danger Certification of hydrogen supply and explosive zone by Technical Inspection Agency

This table includes only aspects particularly relevant for research and for translational MRI, i.e., those which go beyond conventional clinical MRI system siting.

Besides legal and regulatory restrictions, further requirements typically not implemented in a conventional clinical MRI setting need to be fulfilled for acceptance of a dual use MRI system by physicians, researchers, and volunteers or patients:

(i) improved hygiene rules, in particular for zoonosis prevention, i.e., the infection of humans with pathogens from animals and vice versa,
(ii) prevention of smell nuisance due to pig scanning prior to human scanning, and
(iii) prevention of direct contact between humans and animals.

These aspects needed special attention both on structural works of the building, and organizational measures.

Measures for vandalism protection have been implemented, e.g., no facility markings or publicly available facility plans exist.

#### 2.2.1. 7 T Magnet room

#### 2.2.2. Infrastructure for imaging of human subjects

**Figure 1:**
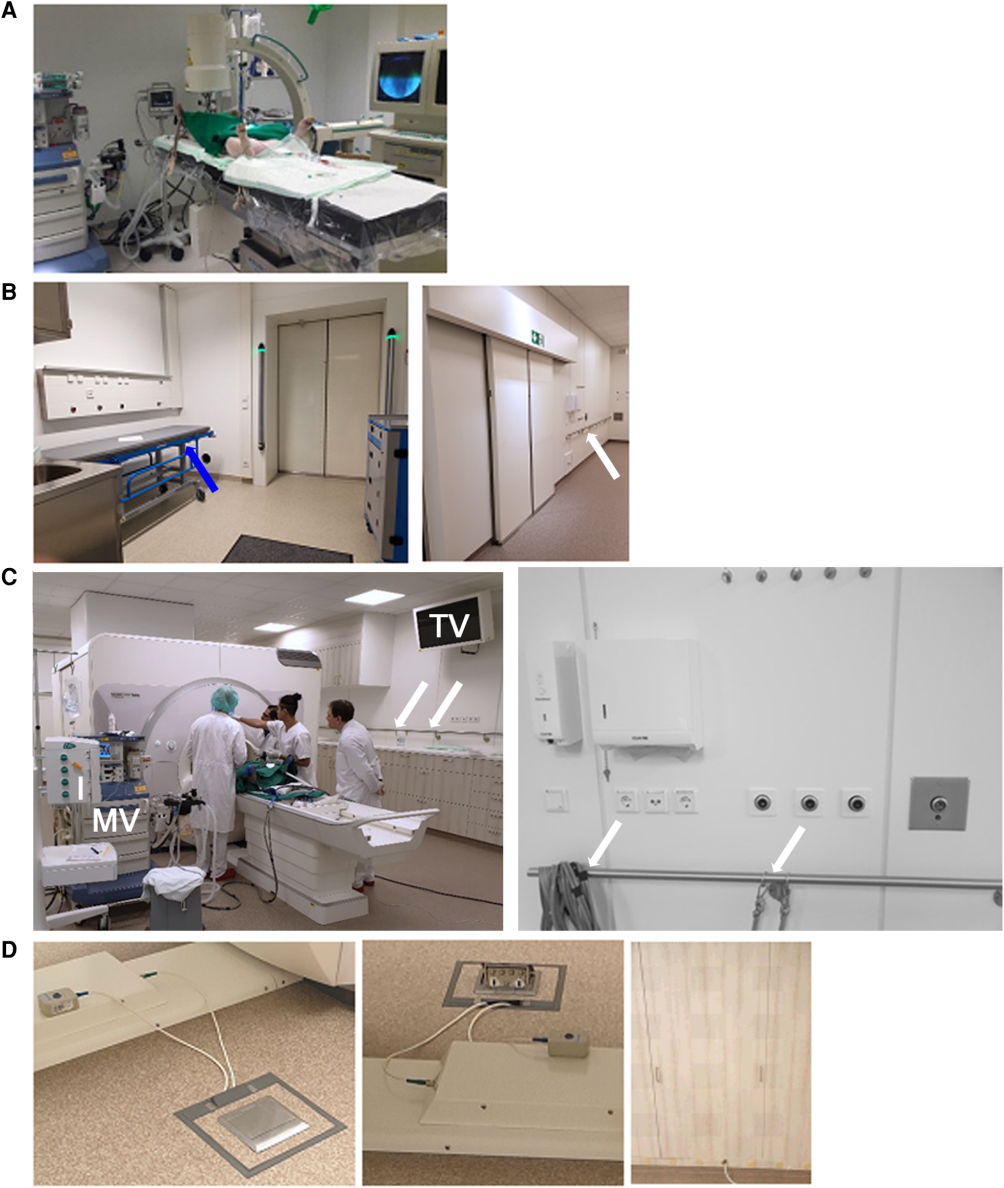
Key components of the translational MRI infrastructure. (**A**) Operating theater dedicated to large animal surgery, including a used C-arm x-ray fluoroscopy machine for monitoring of instrumentation of the animals, e.g., balloon-occlusion for induction of myocardial ischemia or infarction. Radiative shielding of the room's walls was implemented to fulfill legal requirements for x-ray scanning. Moreover, Doppler ultrasound allows for further characterization of the animals. (**B**) The animal waiting area is adjacent to the magnet room. Animals are positioned on a magnetic trolley (blue arrow), on which the animals can be transported through the sliding door and magnetic material detector to the magnet room. Access to pressurized air, and anesthesia gas extraction system allow for mechanical ventilation of the anesthetized animals before they are granted access to the magnet room through the magnet detectors (vertical, with currently green lights on top). To reduce anesthesia duration and animal stress, every attempt is made to keep this phase as short as possible. (**C**) Magnet room during animal MRI, including MR-compatible mechanical ventilator (MV), MR-compatible infusion machines (I), and an in-room monitor (TV) for display of physiological monitoring signals for the operators of the animals. Stainless steel fixation structures (arrows) are installed on all walls. They allow fixation of instrumentation using cords to prevent magnetic attraction and the subsequent risk of accidents hurting human subjects, personnel, animals, or damaging equipment. (**D**) Cable canals below the floor and openings in cabinet doors allow experimental setups with low risk for tripping hazard risk.

For the implementation of the dual use concept, a key requirement was the prevention of accidental direct contact between human subjects undergoing a 7 T measurement, and an animal. Therefore, access routes to the magnet room for human subjects and animals are completely separated [c.f., requirement (iii) and Figure 1]. Access for humans is like that in a conventional clinical 7 T MRI installation comprising of a waiting area, changing rooms where the subjects undergoing an MRI are supplied with amagnetic clothes, a preparation room with an MRI-compatible adjustable-height amagnetic patient trolley. On our request, the trolley was certified “MR Conditional” for 7 T use (MR5501, Wardray Premise, Thames Ditton, Surrey, United Kingdom). Access to the magnet room is through a metal detector (ferroguard Assure, Imaging Solutions, Knobel Court Shailer Park, Queensland, Australia). Since the patient table of the UHF-MRI system cannot be moved down to allow easier access for human subjects, an additional amagnetic step ladder with guard railing (RCN Medizin- und Rehatechnik GmbH, Sargenroth, Germany) is available as well, in particular for mobility impaired human subjects.

In cases of emergency events during the MRI examination, the patient needs to be removed from the MRI system's patient table on the previously mentioned amagnetic trolley using a rollboard. In the patient preparation room, a defibrillator (AED Plus, ZOLL Medical Deutschland GmbH, Cologne, Germany) and emergency equipment according to the hospitals guidelines for cardiopulmonary resuscitation are available. Measurements in patients with suspected or known cardiovascular disease are performed in the presence of a physician with training in cardiopulmonary resuscitation.

#### 2.2.3. Infrastructure for imaging of large animals

For animals, access from the operating theater (Figure 1A) to the magnet room is *via* an access route completely distinct from the one for human subjects. It is directly connected to the CHFC's large animal infrastructure comprising large animal housing, large animal operating theater, and further supporting infrastructure. Anesthesia, instrumentation of the animals, and potential experimental procedures before UHF-MRI are performed in the large animal housing area and operating theater (Figure 1A). There, an older C-arm x-ray machine was available during this study for real-time control of interventional procedures such as balloon occlusion of coronary arteries. Soon, a latest generation C-arm x-ray machine with flatpanel detector (Ziehm Vision FD, Ziem Imaging GmbH, Nuremberg, Germany) machine will be available for better control of the intervention. Subsequently, the animals are transported under conditions of manual ventilation to the MRI using a dedicated nonmagnetic transport trolley and a separated elevator accessible only for authorized personnel. To streamline human and animal use, a special waiting area has been implemented which allows animals ready for an MRI scan to wait under controlled and well-ventilated conditions (Figure 1B) until human measurements are finished and the animals can be brought into the magnet room. In the waiting area, medical oxygen, pressurized (5 bar) oxygen and air and, vacuum for anesthesia gas extraction removal by suction are available to allow stable animal ventilation and anesthesia before the MRI scan. The animal is shifted to another amagnetic movable table (MR5501, c.f. above) dedicated only for animals, on which the animals can later safely access the magnet of the scanner. To be identifiable as an amagnetic device, the animal trolley is colored with an eye-catching blue color and an “MR safe” green label (c.f., Figure 1B).

During a human MRI scan, a sliding-door between the animal waiting area and the magnet room is closed permanently. The door can be opened on request from the magnet room, allowing animal and personnel access to the UHF-MRI system through another metal detector (ferroguard Assure, c.f. Figure 1B). This second metal detector aims at preventing of the risk of transporting magnetic instruments from or for experimentation into the magnet room, e.g., gas bottles, magnetic instruments such as scissors and knives. Once all patients have left the magnet room and patient access doors are closed, the sliding door between the animal waiting area and the magnet room is opened and the animal is rolled to the MRI system using the amagnetic trolley. During the MRI exam the sliding door (and the patient access door to the magnet room) needs to be closed to provide the necessary RF-shielding of the UHF-MRI system.

For animal experiments, the equipment shown in Table 3 was available. In humans, no anesthesia is performed. It is important to note that no equipment certified for 7 T use was available. Therefore, proper functioning within the magnetic and electromagnetic fields of the MRI system was assured by keeping the equipment outside the 0.5 mT (5 Gauss) line of the 7T MRI system's stray field, which is marked on the floor. Moreover, proper functioning of the MRI system with the devices running was also tested to prevent from electromagnetic interference degrading the MRI measurements.

**Table 3:** Equipment available for large animal Experimentation.

Purpose	Equipment Available	Remarks
**Mechanical ventilation**	**Fabius MRI, Dräger, Lübeck, Germany**	**MR-compatible (≤3 T) ventilator**
**Monitoring of vital signs**	**M3 Tesla, Dräger, Lübeck, Germany**	**MR-compatible (≤3 T) patient monitor**
Monitoring expiratory CO_2_ following repeated breath holds	N-85, Nellcor, Covidien, Mansfield, USA	Handheld capnograph
Monitoring rectal body temperature	Optocon AG, Dresden, Germany	Fiber-optic sensor thermometer
**Monitoring blood oxygen saturation (spO_2_),** ${\mathrm{spO}}_{2}$**-derived heart rate**	**8600FO, Nonin Medical Inc., Plymouth, USA**	**Pulse oximeter**
**Hydration of animal and application of drugs**	**Space® infusion pumps**	**Installed within MR safe housing (≤3 T): Space Station (Braun, Melsungen, Germany)**
**Remote controlled contrast-agent administration**	**MEDRAD® Spectris Solaris EP, Bayer AG, Leverkusen, Germany (0.01–10 ml/sec)**	**Programmable injection system**
Warming-system to keep body temperature at normal levels	Hico aquaterm 660, Hico Medical Systems, Cologne, Germany	

**Bold-marked devices** are certified for use in magnetic fields up to 3 T. No devices certified for use at 7 T magnetic field were available. In all devices, adequate functioning was tested before use in animals. Moreover, risk from mechanical attraction to the magnetic field of the MRI scanner was leveraged by tie-down straps fixed at the scanner room's walls (c.f., Figure 1C).

During experimental research, often additional devices need to be used which were not designed for use in an (ultrahigh field) MRI environment. They typically are not MRI-compatible or need to stay outside of the scanner room because of the danger of mechanical attraction by the MRI system's strong static magnetic field, potential radiofrequency interference between the device and the MRI system, or to keep the scanner room free from unnecessary devices causing potential safety hazards. Examples for such devices are mechanical ventilators, liquid-driven heating blankets, or gas bottles intended to provide special gases for experimental purposes. In our installation, these devices can be left in the waiting room with closed sliding doors. Waveguides allow for radiofrequency-safe access to the magnet room by non-metallic tubes. To allow for access to the magnet room using DC, low- or high-frequency electric signals, filter plates with corresponding electrical filters are available from three sides of the Faraday cage.

If devices need to be used which may experience mechanical attraction by the strong magnetic field of the UHF-MRI system, mechanical fixation can be provided by nonmagnetic stainless steel surrounding the whole interior of the Faraday cage. Tie-down straps between the fixation structures and the devices allow for prevention of unintended, mechanically induced motion of devices into the magnet bore and, thus, ensure safety of the personnel and devices during the experimentation (Figure 1C). Within the magnet room, coat hooks allow dropping of clothing worn during the experiment.

Access to medical oxygen, pressurized (5 bar) oxygen and air, and anesthesia gas exhaust are available from two sides in the magnet room. Oxygen and air are delivered from gas bottles outside the magnet room. Cable canals below the floor of the magnet room prevent from tripping hazards from gas tubes or electrical wires. They run from two filter plates on two sides of the magnet room to a position next to the UHF-MRI system (Figure 1D).

To prevent smell nuisance of personnel and human subjects following pig imaging, air-condition in the magnet room facilitates more rapid air exchange than in a clinical installation (12 times/h instead of 8 times/h in normal MRI environments). The animal's odor nuisance typically is removed within a few hours.

Disinfection of an MRI system for the purpose of zoonosis prevention may impose further constraints regarding disinfection requirement usually implemented in clinical MRI systems. Therefore, consultation with the MRI system's vendor was essential to define a disinfection procedure for zoonosis prevention compatible with the MRI systems hardware: disinfection of the MRI system and scanner room, and used hardware (including RF coils) is performed after animal experiments and before the next human examination using a broad spectrum surface disinfectant [Incidin® Plus 0.5%, Ecolab Inc., St. Paul (MN), USA] which has been proven to be effective against bacterial and fungal pathogens (36). Protective plastic covering is used in all large animal measurements to prevent any body fluids (e.g., blood, urine), excrements, or ruminant substances from getting into direct contact with the surface of the MRI system. Moreover, all hardware is used either in animals or in humans, and consumables for animals and humans are stored in different places.

In experimental imaging, typically strong gradients may be used during extraordinary long scan times of several hours which may introduce acoustic disturbance outside of the UHF-MRI area. Although acoustic emission of the latest technology UHF-MRI systems is largely reduced compared to earlier technology, the space between the Faraday cage and the surrounding building walls has been filled with soundproofing wool. In addition, an oblique aluminum water shield has been positioned above the Faraday cage to prevent water reaching the Faraday cage and magnet in the event of water damage above the UHF-MRI installation. The shield redirects water to the side of the Faraday cage where it is drained by the normal hospital's infrastructure. An electronic hygrometer, which is connected to the hospital's electronic bus system, creates an alarm in the case of water reaching the shield anyway. Personal experience of the main author of this study at different public organizations showed that such events are not completely unlikely.

For work with genetically modified animals the MRI system is also certified as a biosafety level S1 area according to the German gentech law. This is feasible because at biosafety level S1 no risk for human health environment is involved. Only very few genetically modified large animal models exist. Therefore, no previsions were made for providing a specific-pathogen free (SPF) housing and experimentation environment. Such an environment would necessitate additional measures to control the hygiene status of the animals, and to prevent infection of the animals by the environmental air or personnel.

#### 2.2.4. Imaging of special gases

Experimental imaging, e.g., using para-hydrogen induced hyperpolarization (PHIP) for metabolic studies (37–39), may include the need to use explosive gases such as hydrogen near or in the MRI system. The safe use requires the peak concentration of hydrogen to always remain below the lower explosion limit. During planning of the UHF-MRI installation it was verified that the airflow from the 12-fold air-condition and that from the UHF-MRI system go in parallel and, thus, the lower explosion limit is never reached in the experimental setup used (40).

Electrical switches may impose a theoretical risk for ignition of gas. Therefore, it was decided to keep all potentially spark-producing electrical switches below the height of the experiment on the patient table, i.e., even if gas would escape from the experiment, it would not reach sparks produced by switching electrical power. Moreover, the planned experimental setup contained a long acrylic glass tube within the UHF-MRI systems bore (40) such that it would be impossible that hydrogen would get into contact with sparks theoretically produced in case of failure during switching strong gradient fields within the UHF-MRI.

#### 2.2.5. Computer infrastructure

The magnet room is equipped with ethernet network cables and connectors with a 100 Mb/s connection to the whole computer network of the hospital (Schwarz Abschirmtechnik GmbH & Co KG, Hennef/Sieg, Germany). Therefore, dedicated measurement computers or physiologic measurement devices in the magnet room can be operated remotely if necessary, and data can be downloaded *via* the network. The magnet room is equipped with a custom-built 7 T-compatible dedicated wall-mounted computer screen placed in an electromagnetically shielded housing (c.f., Figure 1C) and connected to the control room *via* optical cable for the signal transfer (MR Schutztechnik Kabinenbaugesellschaft mbH, Dieburg, Germany). This allows for demonstrating images or physiological signals from the workstations placed in the control room to the researchers or animal care personnel in the magnet room without them having to leave the animal alone.

During animal experiments, different computers may be used in addition to the MRI systems. Although no real-time exchange of information is currently intended, it was considered helpful to synchronize all computers including those from the MRI systems to the network time protocol (ntp) server of Wuerzburg university clinics which is connected to a national Stratum-1 time server. By provision of a unique time base, system times of all computers connected to the ntp-service should be accurate to better than 1 s and in theory might reach the theoretical limit of the ntpv4 protocol of 10 ms or better (41, 42). This is of particular interest when comparing image data with physiologic signals using the UHF-MRI system's physiologic monitoring unit, or the physiologic monitoring device (ADInstruments Ltd, Oxford, UK). The latter is dedicated for animal experiments and provides reliable physiologic signals such as invasive blood pressure, 12-lead electrocardiogram, or peripheral blood oxygen saturation (spO_2_) using external sensors. Physiologic data can be monitored automatically and by the ntp-synchronization direct assignment of physiologic data to an image is feasible.

For rapid file transfer of large image and raw data sets, 10 GB ethernet connection is available between the technical room of the MRI system, the coil lab, and the offices of users of the large image data sets within the building. Moreover, since the technical room is well ventilated, it also contains two of the workgroup's compute servers (currently dual Xeon E5 2,630, 6 cores / 12 threads ×2, 2.4 GHz, dual Tesla K80 GPU, 128 and 512 GB RAM, 28 TB disk space). For automatic storage of the acquired images, a dedicated picture archiving system (PACS) for research data has been implemented to which MRI images are transferred after the measurements.

The described imaging infrastructure will be an essential part in data generation and postprocessing. Data pipelines have been developed comprising of concepts to search for measured research MRI data in analogy to the CHFC's data warehouse system (43) e.g., for use in machine or deep learning research (44, 45). The mentioned PACS system for experimental and human data is distinct from the normal clinical PACS system. All studies, experimental and human, are stored in a pseudonymized fashion comprising a principal investigator identifier, a study identifier, and a study running number,

Currently, efforts are ongoing to connect it to the virtual research environment currently being installed by the Faculty of Medicine in collaboration with the Charité's virtual research environment. This virtual research environment will provide (46) (i) workflows for radiologic imaging data, (ii) a model for cataloguing data to ease finding data according to FAIR principles (47), (iii) workbenches for modelling, simulating, and analyzing data, (iv) a portal that is open also for external users, (v) interoperability with international data commons like those developed under the European Open Science Cloud (48).

To provide direct access to electric signals from the electronic cabinets of the MRI system to users at the MRI console during an experiment or to the coil laboratory where dedicated measurement equipment is available, coaxial cables with BNC and N-type connectors are routed from the technical room over the Faraday cage to the scanner's operating console and the coil lab with its dedicated measurement devices. This allows for direct display of control and measurement of signals available at the electronic cabinets of the MRI system. Thus, only a very limited number of personnel or researchers need direct access to the technical room of the MRI system.

### 2.3. Radiofrequency coil concept

The field strength of the MRI system and the radiofrequency coils used for RF transmission and signal reception are the main determinants for optimum image quality. In research, imaging often is pushed to extreme levels of speed or physical sensitivity. Signal loss due to suboptimal RF coils cannot be recovered later. Thus, the availability of optimal RF coils is an existential prerequisite in an imaging research environment (c.f., Equation 1). Therefore, the in-house RF coil lab developed optimal coils for imaging objects across a variety of spatial scales, from submillimeter cardiac organoids, zebra fish, and excised mouse hearts (49), rodents *in-vivo*, to large animals such as pigs *in-vivo* (50) and excised pig hearts (51). Concepts for human RF coils have been developed as well (26) but not yet applied to humans.

For human imaging several coils are available, in particular a 1Tx/16Rx cardiac coil (MRI.Tools GmbH, Berlin, Germany) which was provided by the MRI system's vendor during MRI installation. Recently, a new circular cardiac 8Tx/16Rx RF coil has become available following a collaboration between Siemens Healthineers (Erlangen, Germany), Rapid Biomedical GmbH (Rimpar, Germany), and authors of this manuscript (52). This coil is now available commercially. It provides better $B_{1}^{+}$ homogeneity than the single transmit (Tx) channel-coil. In the future it will allow the use of $B_{1}^{+}$ -shimming and parallel transmission (pTx) methodology. The latter two techniques are considered necessary requirements for good cardiac image quality although methodologies need to be developed further (25, 27, 53).

**Figure 2:**
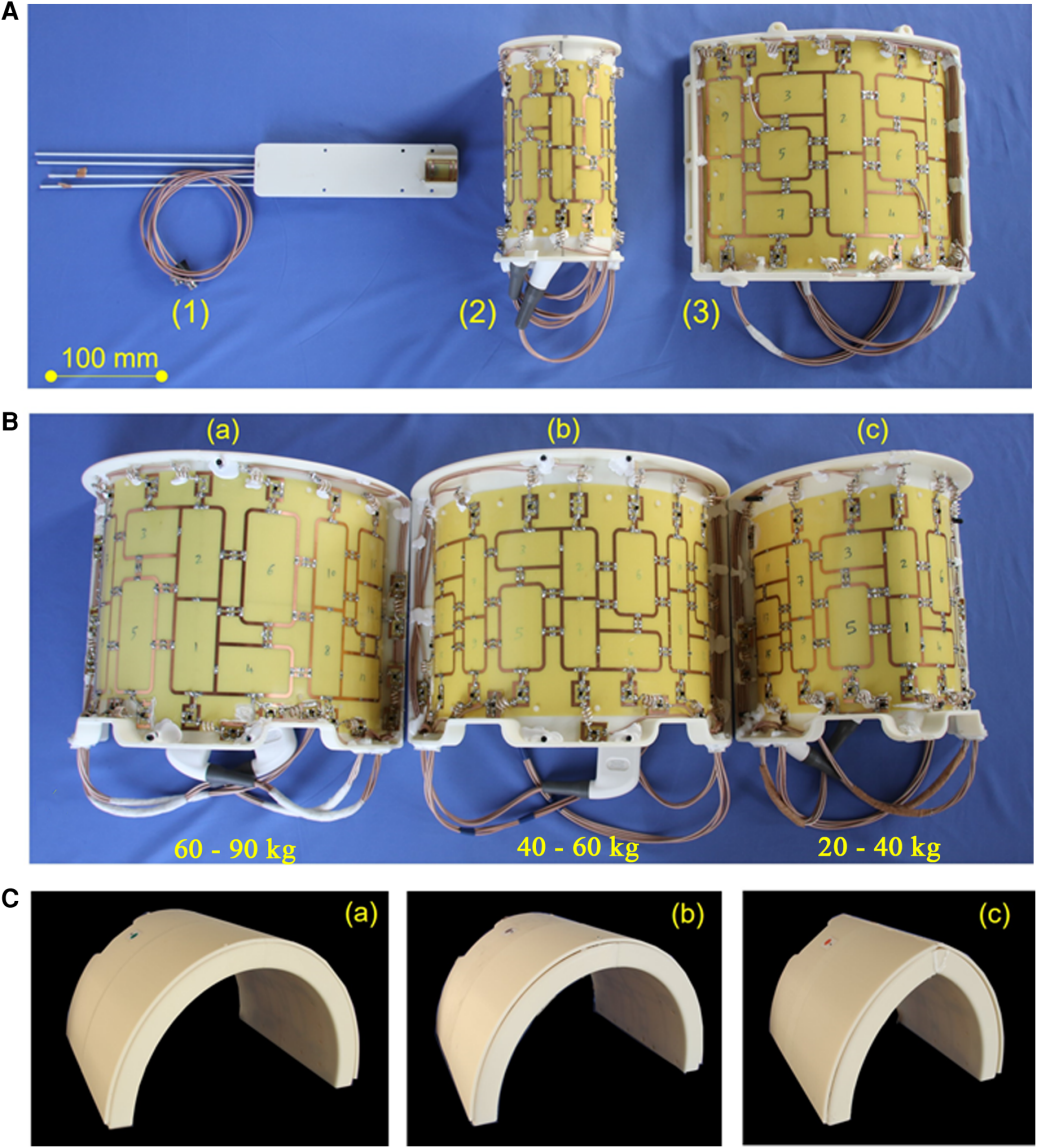
RF coil concept of in-house developed coils for experimental imaging. (**A**): (1) Coil for cardiac MRI in small animals (tunable to the ^1^H and ^19^F Larmor frequency) suitable for mice up to 40 g body weight. This coil is for use with the small animal MRI system. All other coils shown in this figure were developed in-house for use with the whole-body 7 T MRI system: (2) 8Tx/16Rx pTX coil for imaging excised organs from large animals in the whole-body 7 T MRI system (c.f., Figure 5). (3) 8Tx/16Rx array for 7 T cardiac MRI in human subjects. Panel (**B**) Dedicated 8Tx/16Rx arrays for cardiac MRI in pigs of different weights: (a) 60–90 kg, (b) 40–60 kg, (c) 20–40 kg. The shape of each coil is adapted to the typical shape of a German landrace pig. The three differently sized coils allow for optimal coil filling factor and, thus, SNR. The scanner bore size as well as, to a minor extent, the thickness of the RF coils, limit the scanning of pigs to 90 kg or less. (**C**) Visualization of the housing of the three coils in (**B**). The different shape of the thorax of different sized animals is visible. No posterior coil elements were used because their signal contribution for the MRI signal of the heart was typically 5% and, thus, negligible.

Dedicated RF coils for large animal MRI at 7 T are not available commercially. Since 7 T MRI systems include no body-coil for overall RF transmission, all coils need to be transmit-receive coils. Simple flexible receive coil concepts such as those available for clinical imaging are not available This typically results in suboptimal results at 7 T since the shape of a pig's thorax has a different shape compared to that of a human and, thus, both RF transmission and reception would be particularly inhomogeneous. Moreover, a further anatomical consideration in coil development was physiological positioning of the animal's legs during an MRI scan which turned out to be difficult with human coils. Since key to optimal imaging is an appropriate RF coil with a high filling factor [i.e., surrounding or at least being as near to the heart as possible, c.f. (1)], three dedicated RF coils for large animal MRI were developed in-house for different sizes of animals such that for each animal size between 25 kg and 90 kg an optimal-sized pTx coil with optimal RF transmission and reception is available (Figure 2B,C) (14, 15, 54, 55).

For certain biomedical research questions, *in-vivo* imaging is not necessary, and the outstanding image quality and information obtained in imaging of arrested hearts *ex-vivo* may lead to further insights. This is because a lack of motion helps increase the temporal efficiency of the scan process and prevents the development of image artifacts from physiologic (cardiac, respiratory, or peristaltic) motion. Moreover, the RF coil elements are in close proximity to the heart and, thus, a high SNR can be obtained. Since we have found that assessment of tissue microstructure by diffusion or diffusion weighted imaging can be successfully performed *ex-vivo* (49) although effects of fixation may need to be considered if quantitative numbers are desired (56), a dedicated *ex-vivo* coil for high-resolution high-SNR imaging of explanted pig hearts was developed. The 16 elements of this coil array are located on an elliptical cylinder surface with 12 cm long axis to enable optimal $B_{1}^{+}$ profiles for transmission as well as high SNR and low g-factor for signal receive (51, 57).

Mainly for hygienic reasons, but also because of the strategy to optimize the coil's shape to that of the object under study, coils are exclusively used either for human or for large animal imaging.

### 2.4. Imaging protocols

Since cardiac MRI was not a standard product on the whole-body MRI system, all pulse sequences and measurement systems needed to be implemented from scratch, based on existing pulse sequence files and protocols available on the MRI system e.g., for neurological imaging. This also included the definition of localization pulse sequences. In consequence, currently not all imaging protocols feasible at clinical MRI systems are available optimized for UHF-MRI in the heart.

#### 2.4.1. Measurements in pigs at the whole-body MRI system

##### 2.4.1.1. *In-vivo* imaging of pig hearts at 7 T

Large animal measurements are performed in female German Landrace pigs. Since our RF coil concept was designed for bodyweight in the range between 25 and 90 kg, the vendor of the animals (Heinrichs Tierzucht GmbH, Heinsberg, Germany) is advised to select for delivery animals with bodyweight of up to 25 kg. After initial acclimatization for 10 days, the pig undergonesthesia using atropine (0.5 mg/kg), azaperone (8 mg/kg), and ketamine (15–20 mg/kg). Anesthesia is maintained intravenously using propofol (2–10 mg/kg/h) and fentanyl (0.01–0.03 mg/kg/h).

Animals are scanned in the whole-body UHF-MRI system using one of the three mentioned custom-designed 8Tx/16Rx cardiac array coils. An initial study aimed to establish 7 T cardiac MRI in a large animal model of acute and chronic infarction. Therefore, imaging was performed 7 days before induction of myocardial infarction (MI), and at days 3, 14, and 58 after the operation. MI was induced *via* a 90 min balloon catheter occlusion of the proximal left anterior descending artery with subsequent reperfusion. The entire animal use protocol was approved by the local authorities (Government of lower Franconia, Wuerzburg, Germany).

CINE images (30 cardiac phases) were acquired using the same sequences used in human measurements. Only minor adjustments were needed. A continuous short axis stack of 10–15 slices with TE/TR = 2.92/45 ms, FOV = 340 × 318, bandwidth = 893 Hz/Px, and base resolution = 288, leading to an interpolated in-plane resolution of 0.6 × 0.6 × 6 mm^3^. In addition, high-resolution CINE acquisition was performed where the baseline resolution was increased to 400, leading to an in-plane spatial resolution of 0.4 × 0.4 × 6 mm^3^. GRAPPA = 3-acceleration was used for signal reception.

The previously acquired CINE stack in short axis orientation was used for localization of subsequent T2* imaging, first pass perfusion imaging, and LGE imaging before/after cardiac arrest. Slice positioning was performed such that one slice was positioned at the level of infarction:

$T_{2}^{*}$-measurements were performed using cardiac triggering by an acoustic system (EasyACT, MRI.Tools) (29). Imaging was performed using a 9-echo gradient-recalled echo pulse sequence with TE-times distributed in the range between 1.1 and 14.6 ms. To account for inevitable $B_{1}^{+}$ inhomogeneities and to obtain a relatively homogeneous overall signal intensity over the heart, the flip-angle was increased by 10% compared to the optimal one found for CINE-acquisition. The volume for *B*_0_-shimming volume was set to fit the visible heart dimensions on each measured slice (22).

Myocardial perfusion was visualized using the first-pass methodology and low dose (∼0.016 mmol/kg bodyweight) gadolinium DOTA (“Gadovist”, Gd molality 1.0 mmol/l) injection, followed by a saline flush. Gadolinium DOTA is the contrast agent often used at our institution in clinical MRI. Two slices per heartbeat were measured within healthy and infarcted myocardium zones. A LGE stack was acquired for scar imaging using an IR-GRE sequence with TE/TI = 1.9/560 ms.

##### 2.4.1.2. *In-vivo* imaging of pig hearts at 3 T

For comparison of CINE imaging at 7 T with 3 T, data obtained in another healthy 40 kg pig at a Siemens Magnetom PRISMA 3 T whole-body MRI system (Siemens Healthineers) were used. Here, imaging was performed using a GRE pulse sequence with TE/TR = 1.37 ms/308 ms, 10° flip angle, bandwidth = 610 Hz/Px and GRAPPA = 2 acceleration. In this measurement, a commercial four element small flex coil was used (58). In prior experiments at that MRI system, this coil was found to provide the best image quality among all vendor-provided human coils in 40 kg pigs. This substudy was approved by the State and Institutional Animal Care Committee (Landesuntersuchungsamt Rheinland-Pfalz, Koblenz, Germany) and conducted according to the ARRIVE guidelines.

##### 2.4.1.3. *Ex-vivo* imaging of pig hearts at 7 T

For optimal visualization of the scar, and for assessment of the influence of physiologic motion on image quality, high-resolution scar imaging (0.4 × 0.5 × 6 mm, 4 averages) was performed at the whole-body MRI system using the previously described LGE pulse sequence after cardiac arrest. For this purpose, cardiac arrest was induced by injection of 150 mg/kg pentobarbital. Subsequently, MRI was first performed *in-situ*, i.e., under identical conditions like the *in-vivo* examinations but without physiologic motion.

Second, further imaging in excised hearts was performed to further increase the image quality. For this purpose, fixation of porcine specimen was achieved *via* immersion in 10% neutral buffered Formalin within 3 h of cardiac arrest. Atria from all hearts were removed to ease the removal of trapped air and fixative distribution, allowing its penetration into the myocardial wall from both sides. In a prior study we have shown that T2 and T2* initially decrease following immersion fixation using formalin, and then increase again for prolonged storage in formalin (56). To provide sufficient time for tissue fixation, MRI scanning is typically performed at least seven days after immersion. Fixed hearts were placed in a plastic container and the sample position was fixed using Fomblin soaked sponges. The container was then slowly filled with Fomblin and excess air removed from the sponges and the heart using a vacuum desiccator. Previously published data suggested that MRI parameters such as *T*_1_, $T_{2}^{*}$, FA, and ADC are stable for many hours of cardiac arrest. Starting the fixation process within three hours post cardiac arrest is thus early enough to prevent influences of tissue degradation.

Because of the large body size, *ex-vivo* imaging *in-situ* was performed using the largest of the large animal coils described in section 2.3 and shown in Figure 2. *Ex-vivo* imaging of excised pig hearts was performed using the 16-element *ex-vivo* coil mentioned at the end of section 2.3. Morphological imaging of excised hearts was performed with TR/TE = 9/3.8 ms, FA = 15°, voxel size = 0.4 × 0.4 × 5 mm^3^, matrix = 256 × 256. *B*_1_-shimming was performed using scanner-side pTX *B*_1_-shimming platform based on the acquisition of *B*_1_-mapping (59). A turbo spin-echo pulse sequence with 16 signal averages was used for ultra-high-resolution imaging of excised pig hearts using an echo train length of 4, TE/TR = 15/2,000 ms, acquisition matrix of 960 × 810, slice thickness = 1 mm and 0.8 mm with a resulting in-plane voxel size of 0.1 × 0.1 mm^2^.

Assessment of myocardial microstructure at ultimate resolution was performed using 2D *ex-vivo* diffusion tensor imagingwith Stejskal-Tanner diffusion encoding to acquire whole heart diffusion tensor images with an isotropic spatial resolution of 0.8 mm. Further parameters for these short-axis acquisitions were: TE/TR = 56/73 ms, raw data matrix = 128 × 104, FOV = 243 × 300 mm, and an GRAPPA acceleration factor of R = 3, 30 diffusion directions (b = 2,000 s/mm^2^), averages: 50, bw = 1,414 Hz/px. Total acquisition time was 3 h 40 min.

Experimental imaging in explanted hearts included very long scanning with durations on the order of several hours. This may impose stress on the hardware of the MRI system well extending beyond that in clinical situations, and it may also include atypical object and gradient orientations otherwise not used in clinical imaging. Therefore, after consultation with the vendor of the UHF-MRI system, it proved to be essential to verify that no harmonics of mechanical resonances of the gradient system would occur in pulse sequences with extensive use of gradient switching, and with durations much longer than those occurring in typical clinical settings. For this purpose, a dedicated software application was developed. Based on confidential information by the vendor, limitations of gradient strength, slew rate and gradient orientation are checked before a new pulse sequence or measurement protocol are started.

#### 2.4.2. Imaging at the experimental MRI system

##### 2.4.2.1. Mouse hearts

Although not the primary aim of the manuscript, we describe here also some exemplary measurements from small animal translational imaging using the Bruker PharmaScan 7 T experimental MRI system of the described infrastructure. *In-vivo* imaging of small animals (mice), cardiac cell organoids, and *ex-vivo* imaging of mouse hearts was performed at room temperature using the proton channel of an inhouse built dual-tuned 1H/23Na Tx/Rx coil (c.f., Figure 2A), or using a liquid-nitrogen cooled vendor-provided 1H mouse heart coil. Cardiac MRI measurements in mice were performed with vendor-provided pulse sequences using prospective triggering by the vendor-provided monitoring system for ECG and breath control (SA Instruments Inc., Stony Brook, NY, USA). Typical animal weight is 30 g. A gradient-recalled echo CINE pulse sequence with the TE/TR = 2/10 ms, matrix size = 132 × 192, reconstructed in-plane pixel resolution = 0.2 × 0.2 × 1 mm^3^, 4 signal averages, and up to 15 cardiac phases (depending on the animals' heart rate) was used.

Anesthesia in mice was performed using isoflurane vaporizers (Northern Vaporizers LTD, London, United Kingdom). Two identical devices are installed, one in the small animal preparation room and one in the Bruker scanner room. The vaporizers are supplied by oxygen from the building's medical gases supply system using standard wall connectors. The animals are kept under isoflurane anesthesia during the whole preparation stage and MRI measurements. The level of isoflurane varied between 2% and 3% depending on the monitored animal's physiological conditions. The animal's body temperature was monitored by an intrarectal sensor connected to the monitoring and gating system. ECG and breathing rate were monitored continuously using golden-platted disk electrodes and a breathing pad sensor, respectively. All sensors were supplied by the monitoring system's vendor. Warming of animals during the whole scan was performed by the vendor-supplied warming pad connected to the water thermostat.

*Ex-vivo* diffusion tensor imaging of mouse hearts was done at our institution as a *remote imaging* project in cooperation with the Charité, Universitätsmedizin Berlin, and the German Centre for Cardiovascular research (DZHK). In Berlin, hearts were excised and fixed (perfusion fixation using formalin) following the *in-vivo* part of the study protocol. Hearts were then sent to Wuerzburg for high resolution (100 µm isotropic) *ex-vivo* DTI at the Bruker MRI system. Further details regarding measurement setup and data analysis can be found in respective publications (60–62). The fixation setup is similar to that used for zebrafish imaging and is described in detail in Supplementary section S1.

In another study, high spatial resolution MRI of the aortic arch in an unfixed mouse *ex-vivo* was performed with a similar diffusion tensor pulse sequence with 80 µm isotropic spatial resolution.

##### 2.4.2.2. Excised pig aorta

To demonstrate the potential of excised-tissue imaging in vascular research, high-resolution imaging and imaging of passive substance diffusion into the vascular wall was performed.

Measurements were performed using a dual channel TX/RX 2.5/3 cm int./ext. diameter, 4 cm length ^1^H-Cryoprobe. Morphologic imaging of an excised porcine aorta was performed with a high-resolution RARE pulse sequence with TR/TE = 6,000/39 ms and with 30 × 30 × 15 µm^3^ voxel dimensions. Scan time of this pulse sequence was 60 min.

Oxidative stress is an important feature in many diseases. TEMPOL with its unpaired single electron can be used to measure the presence of reactive oxygen species (ROS) in oxidative stress. TEMPOL is paramagnetic and, thus, well visualized using T1-weighted imaging. Moreover, it acts as an ROS scavenger, and in that process paramagnetism is lost. Thus, monitoring of the TEMPOL MRI signal can be used as a surrogate marker for the presence of ROS and, thus, oxidative stress (63).

To demonstrate this contrast principle, a 10 mm long vascular specimen extracted from a porcine aorta was monitored during immersion in a 30 mM TEMPOL using semidynamic high-resolution (0.12 × 0.16 × 0.3 mm^3^) T1-weighted RARE imaging (TR/TE = 100/4 ms). To simulate the presence of ROS, after 40 min the vascular specimen was exposed to a 20 mM solution of ascorbic acid. Semidynamic imaging was continued during that exposition as well.

##### 2.4.2.3. Cardiac organoids

Cardiac organoids are of interest to reduce the number of animal experiments [“reduce” of the “3R” concept (64)]. Therefore, a particular interest was to demonstrate MRI of cardiac organoids. The organoids were produced with 2.5 × 10^6^ cells used for seeding by the Institute of Anatomy of Wuerzburg University (details in Supplementary) and transported at physiologic temperature to the Bruker MRI system. Measurements were performed using a dual channel TX/RX 2.5/3 cm int./ext. diameter, 4 cm length ^1^H-Cryoprobe. Cardiac organoids were kept at physiological temperature and scanned immediately after removal from the cell culture. After removal from the cell culture the organoids stopped spontaneous contraction. Therefore, no triggering of the MRI measurement was needed. Cardiac organoids with a diameter of 0.9–2 mm were scanned in 100 µl CBM buffer in a 1 ml sealed syringe for no longer than 50 min to avoid oxygen and glucose tension and temperature shock.

As an application example, organoids were scanned in the native state and exposed to 15 mmol TEMPOL used as specialized contrast agent for studying oxidative stress. MRI was performed using a TurboSpin-Echo pulse sequence with TR/TE = 323/10 ms, *α* = 90°. With a raw data matrix of 160 × 140 and field-of-view 7 mm × 7 mm, the pixel-size was 0.2 × 0.2 × 0.3 mm^3^. More experimental details of TEMPOL imaging are found in Supplementary section S2.

##### 2.4.2.4. Zebrafish

The zebrafish Danio rerio is another important animal model, in particular in cardiovascular genetics. Imaging of zebrafish was performed *ex vivo*. For this, adult Tüpfel longfin zebrafish (ZDB-GENO-990623-2) were euthanized by immersion in a lethal MS222 solution (>0.3 mg/ml in buffered fish water, Sigma-Aldrich, immersion time at least 15 min). After loss of reflexes, a small incision was made to open the abdomen along the ventral midline between the anal pore and the gills. To ensure complete and uniform fixation, zebrafish were fixed in 4% paraformaldehyde (Sigma-Aldrich) on a rocking shaker for at least 72 h at room temperature. MRI was performed subsequently using an in-house 3D-printed fixation device. Housing and husbandry occurred in the aquatic facilities at the hosting institute in accordance with the guidelines of the German animal welfare law and were approved by the local government of Lower Franconia. The fixation setup is described in detail in Supplementary section S1.

#### 2.4.3. Cardiac MRI in human subjects

Human cardiac MRI measurements were performed using vendor-provided cardiac triggered GRE pulse sequences. Details of the setup and pulse sequences have been described in an earlier manuscript (23). In brief, for triggering of imaging to cardiac motion both the scanner integrated ECG system and a third-party acoustic triggering system ACT (MRI TOOLS, Berlin) were available to obtain optimal ECG-signal. Shaving of the chest hair was performed if necessary, and subsequently Nuprep gel (Weaver and Company, Aurora, Co, USA) was used to improve the electrical contact between the ECG electrodes with the body. If ECG-triggering did not result in an adequately good triggering of the measurements, the acoustic triggering system was used.

Sequence parameters for CINE image acquisition were optimized experimentally to achieve optimal SNR and blood-to-tissue contrast in the myocardium (TR/TE = 59/3.6 ms, image matrix = 288 × 228, FOV = 360 × 360 mm). Parallel receive technology (GRAPPA) was used with an acceleration factor of R = 3. The flip-angle (FA) was varied to adjust optimal blood-tissue contrast with a minimal number of artifacts caused by blood flow.

A commercial 1Tx/16Rx coil (MRI TOOLS) was used in the establishment study (23) and subsequently it was replaced with a prototype 8Tx/16RX coil (65) used in single Tx mode with cohort-specific hardware based $B_{1}^{+}$-shimming (66). Finally, the product version of the 8TX/16Rx array was tested for cardiac MRI using “pTX compatibility” mode of the Terra scanner (67). Since the UHF-MRI system is not FDA or CE certified, this coil is also not certified for human use. However, a statement according to article 12 of EU Council Directive 93/42/EEC of June 14, 1993, stating electrical safety and compatibility with the MRI system, was available.

All human measurements were performed after approval was obtained by the ethics committee of the Medical Faculty of the University of Wuerzburg (7/17-sc, 3/19-me). Subjects provided written informed consent to participate in the study.

### 2.5. Organizational concept for dual use imaging

Since a dual use UHF-MRI infrastructure imposes additional challenges and constraints compared to normal clinical MRI installations, we describe here the additional organizational measures which are necessary to ensure a safe research operation.

During pig experimentation, and in experimental imaging of arrested and excised pig hearts, most safety features of the UHF-MRI system were switched-off to allow for use of the full power and technical capabilities of the UHF-MRI system. Only hardware watchdogs remained in effect in these measurements to prevent from damage of the UHF-MRI system's hardware. Particularly, this meant that restrictions for maximum SAR and peripheral nerve stimulation were not effective in pigs. To guarantee the animal's well-being under these conditions, its vital parameters were monitored continuously. Before the next human measurement, all safety systems were reactivated.

#### 2.5.1. Zoonosis prevention

As mentioned before, the concept for prevention of zoonosis from animals to humans comprises of three aspects: (i) prevention from contact with pathogens, (ii) use of different materials for animal and humans, and (iii) temporal splitting of animal and human use such that no human MRI is performed before disinfection after an animal experiment.

The overall concept of our large animal housing is for cardiovascular research. All large animal models are shared within the research groups and drained from the same breeding firm with prior selection to disease free animals. Thus, we assume that animals have the same good health status. Therefore, we assumed the risk for animal-to-animal disease transmission low. Personnel getting into contact with animals wears protective clothing and face masks to reduce the risk of human-to-animal contamination.

Moreover, RF coils, devices, disposables, etc. are either used in animals or humans only. Storage for both is at markedly different places. In addition, measurement time planning respects the key principle that (iii-i) no human subject must be scanned after an animal, (iii-ii) before a detailed disinfection of the MRI system and MRI magnet room was performed by the hospital's cleaning team. The risk of reverse zoonosis, i.e., infection of animals by humans, was considered minimal. Thus, serial scanning of first human subjects and subsequent animal scanning without disinfection in-between is considered acceptable.

In addition, within the magnet room coat hooks (Figure 1C) allow separation between normal clothing and clothing for animal experimentation. Extra disposal receptables were installed for used clothing from animal experiments.

To prevent contamination of the whole-body UHF-MRI system with potentially zoonotic bacteria or viruses, the animals are obtained from a commercial institution specialized in delivery of animals for laboratory use (Heinrichs Tierzucht GmbH, Heinsberg, Germany). Before transporting the animals to Wuerzburg, and a second time after the arrival, animals are tested for *Salmonella* sp., *Yersinia* sp., *Streptococcus suis,* methicillin-resistant *Staphylococcus aureus* (MRSA), carbapenem-resistant *Actinetobacter baumanii*, other carbapenem-resistant gram-negative bacteria, *enterohemorrhagic Escherichia coli* (EHEC), and *Trichophyton mentagrophytes*. Additionally, the pigs are tested biannually for the most relevant porcine pathogens.

Since part of the animal experiments in this study were performed after authorities became aware of the start of the Covid-19 pandemic (68) and since the vendor of animals was located at a national high incidence region (69–71), animals scanned during that time were also tested for SARS-CoV-2 before and after the transport to our institution, although initial reports inclined that SARS-Cov-2 replicates poorly in pigs (72).

To prevent visual contact of a human and an animal, the experimental setup for animal experimentation, including mechanical ventilator, infusion machines etc., is brought in from the animal waiting area only after the human subject has left the magnet room and after the doors from the human subject area including clothing change rooms are closed. After that, the door of the animal waiting area is opened by a switch from the MRI system's console, and animals and experimental devices may be brought to the MRI system.

#### 2.5.2. Human safety

The strong magnetic field of a UHF-MRI systems extend further and creates stronger magnetic forces if ferromagnetic devices are exposed. Therefore, an organizational concept like that described in the guidelines of the German Ultrahigh field Initiative was implemented (73, 74), including mandatory safety training (with yearly repetitions) of researchers and infrastructure (in particular cleaning) personnel. Moreover, non-medical researchers receives dedicated first-aid training which includes risks and measures related to the UHF-MRI system. The organizational concept also requires a physician to always be informed about an ongoing human scan, during which in-house availability is required in case of an emergency. Cardiac patients are only scanned with an UHF-MRI experienced physician being present at the MRI console.

The UHF-MRI system was the first commercial system of the “Terra” series worldwide. Its vendor of the UHF-MRI system provided an EC declaration according to article 12 of EU Council Directive 93/42/EEC of June 14, 1993, stating electrical safety. Since no FDA or CE certification was and still is available currently for cardiac MRI, and since safety-in cardiac UHF-MRI still is an active area of research (28, 75–77), it was decided after consultation with the IRB that exclusion criteria would be kept strict (23) initially in volunteers. In particular, neither subjects with cardiac stents would be scanned initially, nor subjects with other metallic implants such as dental retainers, hip or knee replacement implants, intrauterine devices, or metal-containing tattoos. With increasing availability of safety data and experience (78), exclusion criteria in the future may converge to those of clinical cardiac MRI in patients.

Regarding patient data safety, subject scanning is performed in a pseudonymized fashion i.e., no real patient names are used. Arbitrary patient names used for registering the subjects at the MRI systems comprise of a study-identifier, principal investigator identifier, and running subject number. After the scan, images are transferred to the research PACS system.

#### 2.5.3. Approval procedures

Local authorities may not be familiar with research using UHF-MRI or imaging research in general. Therefore, approval of large animal MRI at the local authorities was also streamlined after consultation with authorities: To achieve this, a veterinarian specialized in laboratory animal science received all related proposals before submission. An overall check was performed if the proposal considers the important aspects of ethics of animal experiments and 3R (Reduce, Replace, Refine) (4, 79–82).

Approval procedures for UHF-MRI in humans can be challenging because IRBs may not be familiar with MRI systems without clearance from FDA or CE, and with UHF-MRI in general. Therefore, a master IRB application was developed and is made available to all local users of the UHF-MRI system so that this master proposal is always up to date regarding general 7 T topics, in particular patient safety. This streamlines IRB application for collaborators and users of the UHF-MRI system since they can focus on the medical aspects of their intended UHF-MRI study.

### 2.6. Data analysis

The analysis of images was performed using in-house developed Matlab scripts (Mathworks, Natick, USA) and ImageJ (National Institutes of Health, Bethesda, MD, USA) software.

Metrics of cardiac function were derived from measured CINE data using CMR software Medis Suite™ (Medis, Leiden, Netherlands) without any post processing of the image data.Image segmentation was performed manually.

Post processing of DTI data included denoising of the data using local principal component analysis as well as motion correction to account for geometrical distortions. Motion correction and tensor reconstruction as well as visualization of fiber tracts was achieved using DSI studio (https://dsi-studio.labsolver.org/, build: Nov 15 2018).

## 3. Results

### 3.1. General UHF-MRI concept

The overall concept, using a clinical whole-body UHF-MRI system in combination with an experimental 7 T MRI system within a hygienically controlled organizational environment to allow for dual use in pigs and humans proved to be successful. More than 100 human cardiac subjects and more than 70 large animal experiments have been performed to date with the whole-body 7 T MRI system. Details of initial studies are not part of the current manuscript and are described elsewhere (14, 15, 22, 23, 44, 49, 52, 54–56, 60, 61, 83, 84) or will be described in future manuscripts (85–87).

### 3.2. Organizational concept for dual use imaging

Research and developments for both pig and human scanning was streamlined by the dual use concept so that the same pulse sequences could be used in either case. Also, measurement protocols, i.e software cards defining specific acquisition parameters for a specific pulse sequence, could either be used directly in both human and pig imaging, or only with minor adaptions to adjust field-of-view and slice thickness in smaller animals compared to adult humans.

Part of the dual use concept is animal testing before transportation of the animal to Wuerzburg. Surprisingly, despite the German landrace pig is an often-used species in and food processing industry, normal stockbreeding vendors did not fulfill hygiene requirements needed for zoonosis prevention. Thus, a specialized vendor for laboratory animals was selected which perfectly complied with our requirements although animals need to be transported over a relatively large distance of 400 km (approx. 250 miles).

Because of the pandemic situation, the 14 pigs which were delivered to our institution in 2021, were all additionally tested for SARS-Cov-2 prior to transportation from the vendors housing facility, and 10 days after arrival at our institution. All tests were negative, no case of zoonosis or SARS-CoV-2 infection has been observed.

The organizational concept was implemented successfully without the need to modify anything. Safety training was well received by researchers and support personnel, in particular also by the cleaning personnel. Receiving extra training, which went well beyond the training they usually receive for cleaning of clinical MRI systems, made them feel involved in this infrastructure. Moreover, cleaning on demand after an animal experiment and before the next human measurement in all cases was well organized, typically in early morning hours, such that no human measurements needed to be delayed. Ventilation of the UHF-MRI magnet room turned out to be very effective and no smell nuisance was observed within a few hours and in particular not at the next morning. Moreover, sound protection proved very well, and no auditory sensation was felt in the lecture hall directly above the UHF-MRI installation despite extensive use of strong and rapidly switched gradients.

The animal waiting area was essential to prevent direct visual contact between human subjects and animals. In practice it turned out that with the current workload of large animal interventions, it is not yet needed to clear the large animal operating theater quickly after instrumentation of a pig before an UHF-MRI measurement. Therefore, it was preferred practice to keep the animal there and transport it directly to the UHF-MRI system as soon as the previous human subject has left the magnet. Still, the waiting area proved to be important because the setup for the upcoming large animal experiment and consumables such as needles, ventilation tubes, etc. can be safely prepared and installed there. This reduces the time to the start of the large animal experiment. In the future, however, when the number of large animal experiments will further increase such that the large animal operating theater needs to be cleared quickly, the waiting area can and will be used as planned in the initial concept.

### 3.3. Cardiac imaging in pigs

#### 3.3.1. *In-vivo* imaging at 7 T

**Figure 3:**
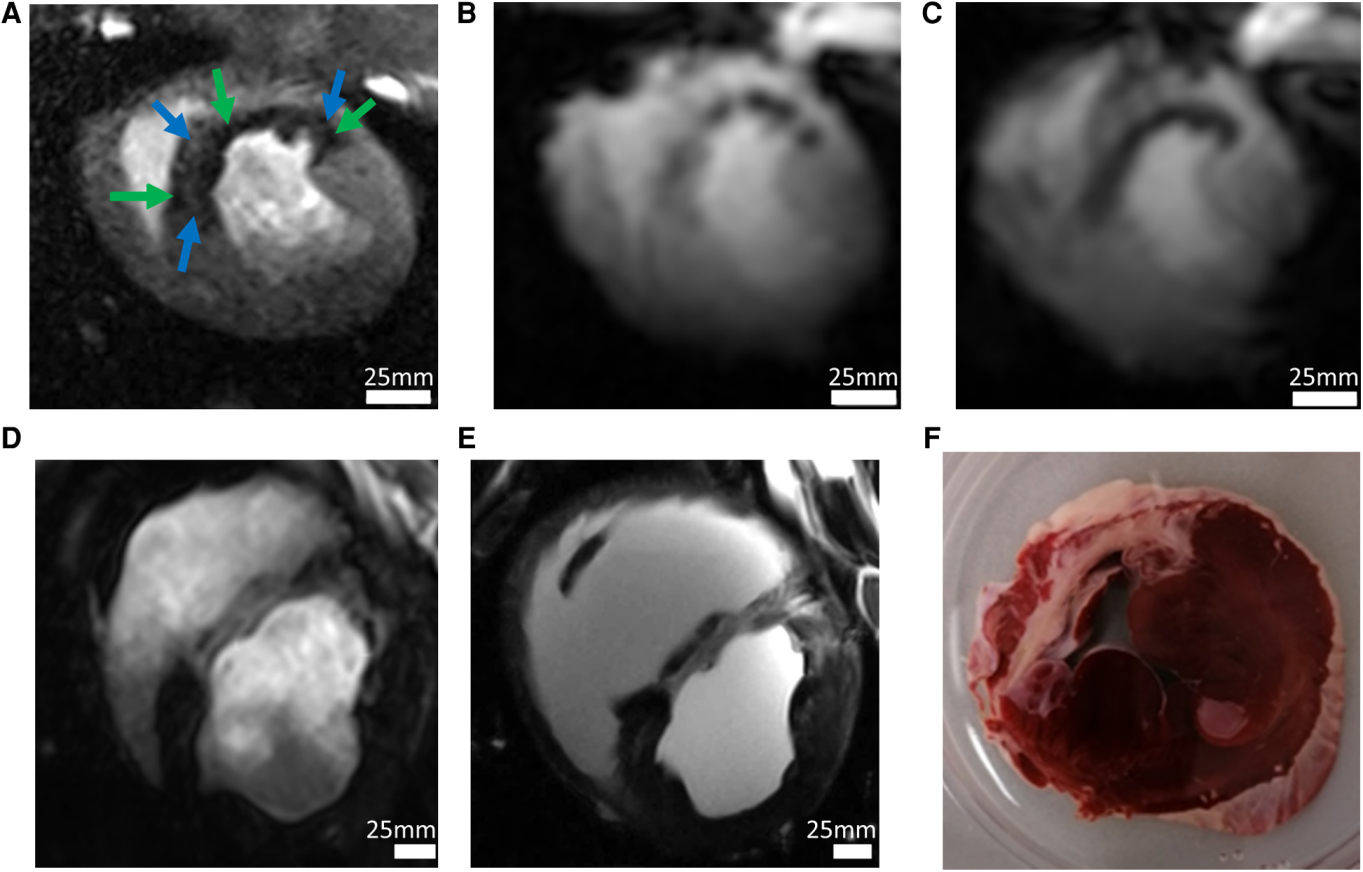
Imaging in a pig model 90 min after occlusion of the left circumflex artery and subsequent reperfusion. (**A**) The high-resolution first-pass perfusion image obtained in the early (day 3) acute phase of myocardial infarction in a short-axis view after injection of 0.0167 mmol/kg BW Gd-DOTA demonstrates a large perfusion defect. Extensive perfusion heterogeneity is observed with hypoperfused myocardium (green arrows). Smaller regions (blue arrows) appear with perfusion almost equal to that of remote myocardium (white arrows). Spatial resolution was 0.95 × 1.4 × 6 mm^3^. (**B**) T2*-weighted imaging at the same examination demonstrates similar inhomogeneities with hypointensities probably induced by acute bleeding. Spatial resolution: 2.2 × 2.5 × 6 mm^3^. (**C**) In the late acute phase, homogenization of T2*-contrast probably demonstrates microstructural changes, e.g., myocardial remodeling. (**D**) High-resolution imaging of chronic myocardial infarction (day 60 after infarction) using the late gadolinium enhancement (LGE) technique following injection of a total of 0.1 mmol/kg BW Gd-DOTA (spatial resolution 1.0 × 1.2 × 6.0 mm^3^). (**E**) LGE imaging *in-situ* 5 min after cardiac arrest. Without physiological motion, a significantly higher spatial resolution (0.4 × 0.4 × 6.0 mm^3^) allows for improved visualization of anatomical details. The *ex-vivo* image is of interest for research questions on structures like scar morphology or tissue microstructure. Alternatively, it may be useful in MRI technology research to demonstrate ground truth of image quality which can be obtained *in-vivo* under conditions of perfect control of motion. (**F**) The histologic section at approximately the same slice location demonstrates the correlation between image signal and tissue histology.

The MRI protocols adapted from human cardiac UHF-MRI for pigs enabled the longitudinal study of cardiac function at acute, sub-acute, and chronic stages of MI in pigs at 7 T (Figure 3). The flip angle was optimized for high blood tissue contrast and, thus, highly reproducible segmentation of the left ventricle and subsequently high accuracy in the derived ejection fraction was obtained. The signal-to-noise ratio and blood tissue contrast were maintained at a high level throughout the study despite increase in animal weight from 33 to 70 kg (Table 4). This demonstrates the excellent image quality which can be obtained in serial animal studies using size-adapted RF coils.

**Table 4:** Results from serial imaging in a pig before and after myocardial infarction.

Measurement	Before MI	4 days post MI	10 days post MI	58 days post MI
Coil #	**I** (25–39 kg)	**I** (25–39 kg)	**II** (40–60 kg)	**III** (61–90 kg)
Pig weight (kg)	37	42	46	75
LVEF (%)	65	41	35	40
LVSV (ml	37	42	32	79
LV mass ED (g)	78	113	117	192
LV mass ES (g)	86	118	132	193

Results from functional analysis: LVEF, Left ventricular ejection fraction; LVSV, left ventricular stroke volume; LV ED, End diastolic LV mass; LV ES, End systolic LV mass.

Figure 3B shows that when compared with 3 T, 7 T delivers not only higher spatial resolution, but also better image quality with regard to overall sharpness of the image detail such as interfaces between the LV and myocardial tissue or papillary muscle. Although this is not a direct one-to-one comparison in a single animal, this data demonstrates the improved image quality at 7 T, which is not only a consequence of the higher field strength but also of the better RF coils available at 7 T.

**Figure 4:**
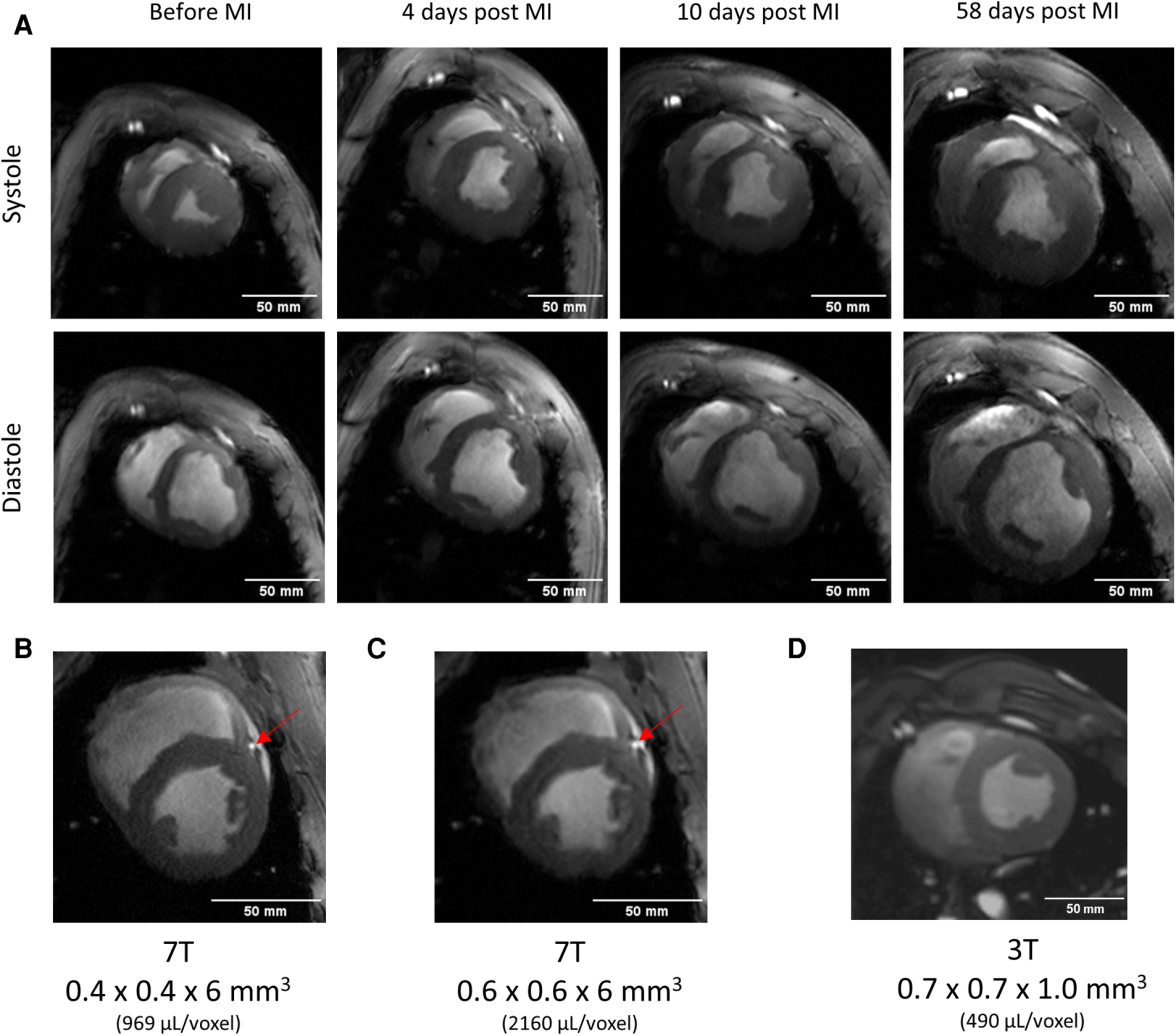
Serial imaging in a pig using the coil concept of Figure 2. Up to 60 days post-infarction. Images were scaled to show the approximately identical size of the heart despite the obvious growth that can be determined from the mass determination given for each measurement. (**A**) High image quality can be obtained throughout the whole series of measurements. Please also note the improved visualization of the left anterior descending (LAD) coronary artery (red arrows) at 7 T. Representative short axis cine image acquired with moderate (**B**) and high (**C**) resolution, and a measurement obtained in another 40 kg pig at 3 T using a commercial four channel flexible coil. (**D**). At 7 T, and with the concept of size- and shape adapted RF-coils, a marked improvement of spatial resolution and image detail is observed when compared to 3 T. For this measurement in another animal, a prior test identified the best-fitting coil which gave the best image quality at 3 T. This was the commercial flexible four-channel coil used for this measurement.

The results shown in Figure 4 demonstrate imaging in a pig model of acute myocardial infarction. High-resolution first-pass perfusion imaging demonstrated a perfusion defect with markedly inhomogeneous perfusion during the wash-in phase of the contrast agent (c.f., Figure 4A). T2*-weighted MRI in early (c.f., Figure 4B) and late (c.f., Figure 4C) acute phase of myocardial infarction demonstrates regions with extensive signal hypointensities, which change from the acute to the late-acute phase. These regions mark regions of delayed or impaired perfusion. Regions with negative $T_{2}^{*}$-contrast mostly correlate with scar regions highlighted by LGE (Figure 4D). However, when compared with areas of positive LGE-contrast the area of $T_{2}^{*}$-reduction was smaller. An interesting and probably essential observation is the substantial correlation of both, localization and size of the $T_{2}^{*}$-contrasted regions with the regions of perfusion deficit detected by the DCE-MRI.

#### 3.3.2. *Ex-vivo* imaging of pig hearts *in-situ*

Figure 4E shows an LGE-image with the arrested heart *in-situ*. Absence of motion allows for a markedly improved image quality and excellent scar delineation. It is obvious that the regions of abnormal signal intensities in first-pass perfusion, T2*, and LGE images spatially correlate within practical limits of slice colocalization with the scar in the histologic section after TTC-staining (Figure 4F).

#### 3.3.3. *Ex-vivo* imaging of excised pig hearts

**Figure 5:**
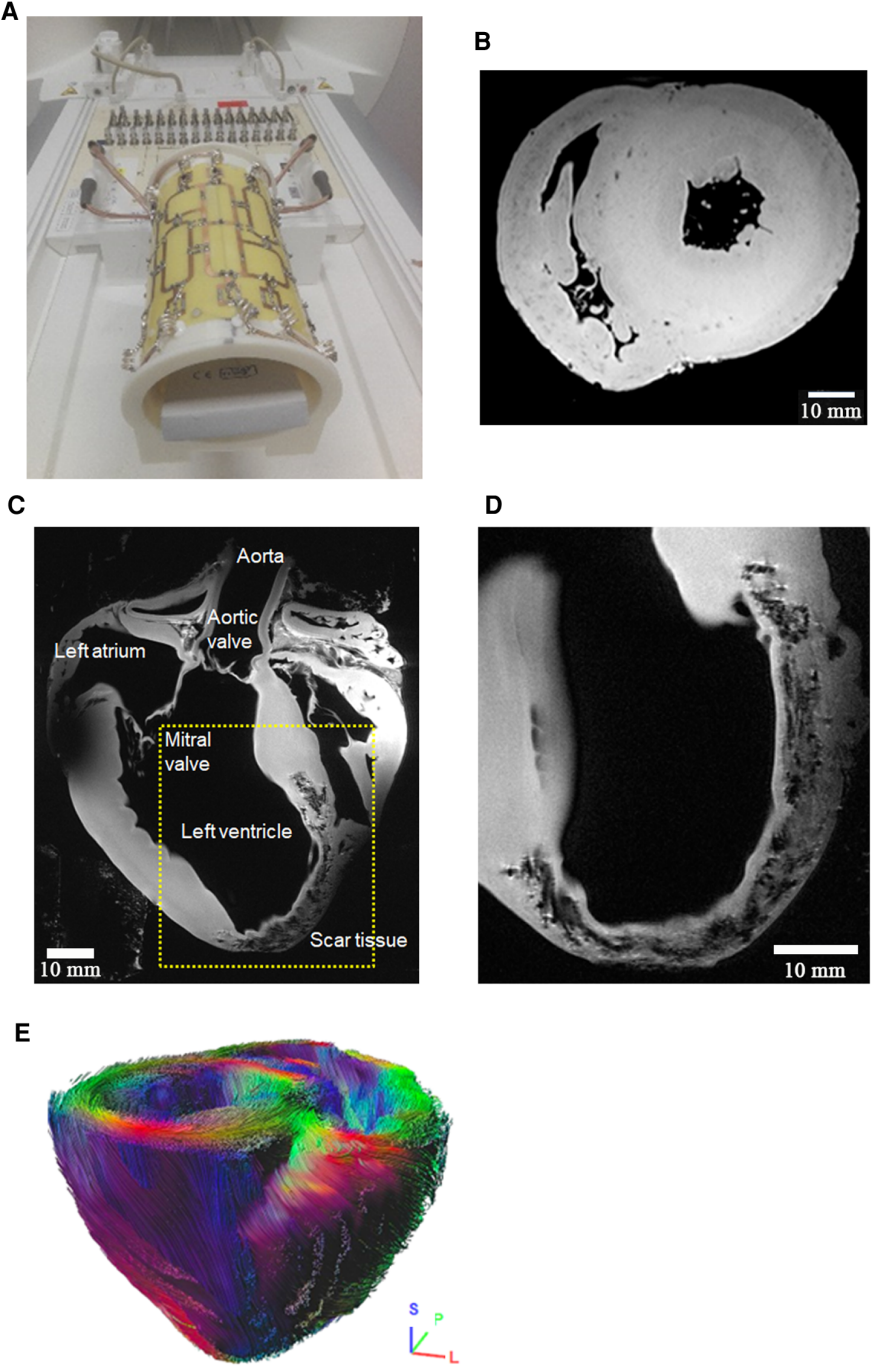
High-resolution *ex-vivo* imaging of excised pig hearts using an (**A**) in-house built 16 element transmit/receive coil. This coil provides an optimal coil filling factor allowing for highest resolution imaging and image quality e.g., in (**B**) high-resolution (0.15 × 0.15 × 0.5 mm^3^) susceptibility weighted imaging in a mid-ventricular short-axis view. Distinct signal intensity variations in the healthy pig heart are depicted in the endo- and epicardial left-ventricular myocardium, and in the right ventricle. The source of this image contrast is not clear at this stage but may be a consequence of transmural variation of the myofiber helix angle, and thus, orientationally variable susceptibility effects in otherwise normal myocardial tissue. Dark dot-like structures denote blood vessels. (**C,D**) Anatomical imaging of late-acute myocardial infarction with 0.1 × 0.1 × 0.8 mm^3^ spatial resolution. The dotted rectangle denotes the approximate position of the magnified portion of the image in (**C**). A *B*_1_-related signal void is seen in the top left portion of subfigure (**C**). Acquisition time for 12 slices was 40 min. Scar tissue structure is well visualized on an almost histologic level. Subfigure (**D**) is an enlarged visualization approximately in the yellow-marked region of (**C**), but at another slice position. High-resolution visualization of infarcted tissue is observed. (**E**) Diffusion tensor imaging-based tracking of myocardial fibers in a healthy pig heart with 0.8 × 0.8 × 0.8 mm^3^ spatial resolution (b = 2,000 sec/mm^2^, scan time 3h:40 min). The visualization shows 25,000 reconstructed fiber bundle tracks. Color-coding corresponds to main eigenvector orientation of the diffusion tensor. Color-coding corresponds to the main eigenvector orientation of the diffusion tensor. The directions are indicated with the small crosshair in the bottom right of the image.

The coil developed for *ex-vivo* imaging of large animal hearts (Figure 5A) enabled proper $B_{1}^{+}$ shimming, leading to increased excitation homogeneity compared to a commercial head coil (1Tx/32Rx Nova Medical), while also on the whole avoiding $B_{1}^{+}$ **-**related signal interferences. In addition, the close arrangement of coil elements resulted in low *g*-factors in the parallel receive technology (*g_R_*_2_ = 1.02, *g_R_*_3_ = 1.06, *g_R_*_4_ = 1.10, *g_R_*_6_ = 1.21) and therefore, improvements in parallel imaging (GRAPPA) acceleration. In comparison, average SNR was improved by ∼75% and more throughout the heart. These improvements enabled high-resolution, high fidelity, *ex-vivo* scans (Figure 5). Susceptibility-weighted imaging (Figure 5B) denotes distinct single vessel signatures within the right- and left-ventricular myocardium. Moreover, a distinct transmural contrast is observed in the susceptibility-weighted image. In the excised pig heart in late-acute phase after myocardial infarction, extremely high spatial resolution was obtained. The complex structure of the myocardial scar is well visualized (Figures 5C,D). Diffusion-tensor imaging (Figure 5E) in a normal pig heart delivers distinct information on the myofiber orientation. Moreover, the well-known variation of the helix angle within the transmural direction can be well visualized.

#### 3.3.4. Experimental imaging for translational research

##### 3.3.4.1. Imaging of mouse hearts and aorta

**Figure 6:**
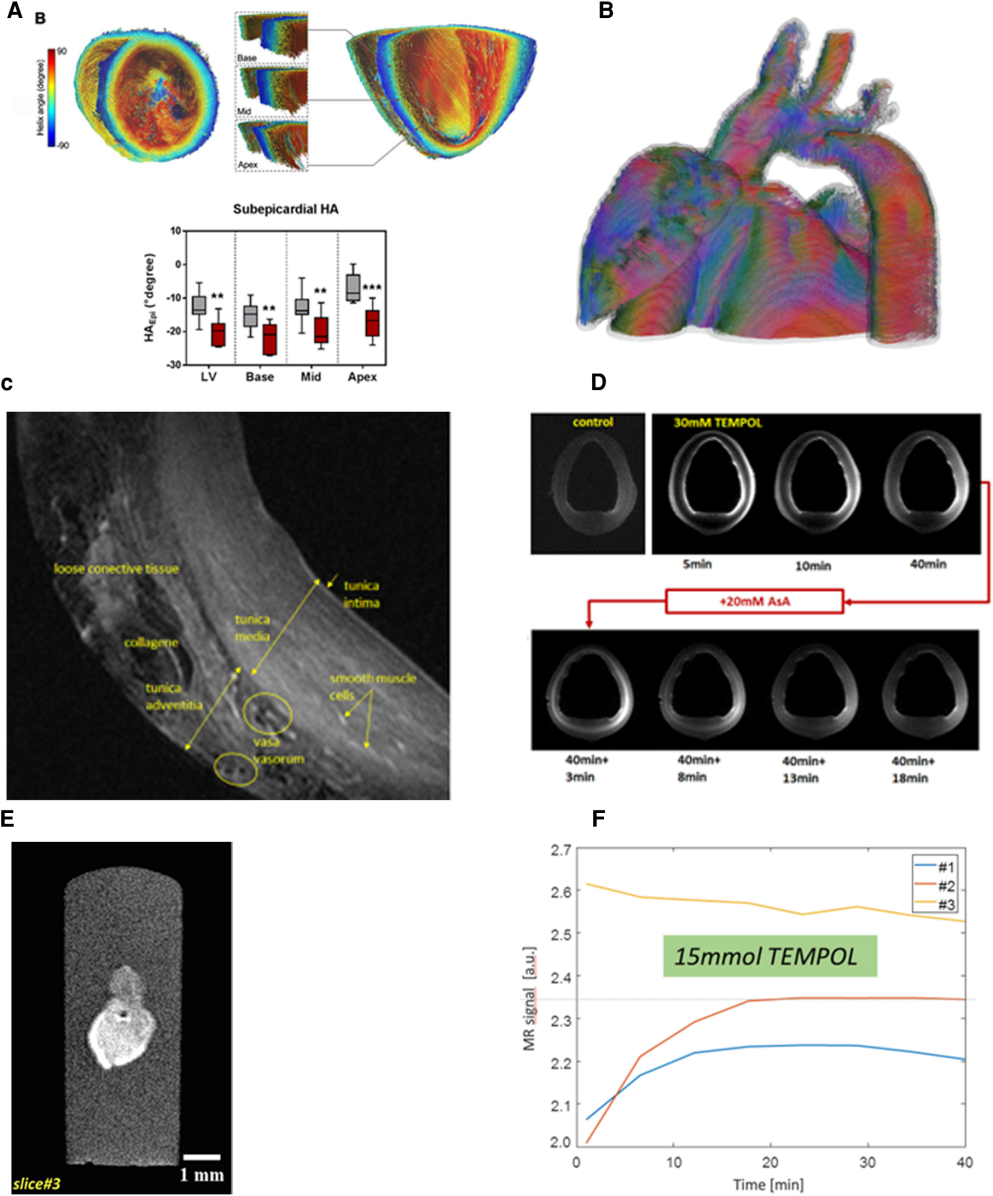
Experimental imaging in excised mouse hearts using a liquid nitrogen-cooled RF coil. (**A**) Diffusion tensor imaging-based tracking of myocardial myofibers with 100 µm^3^ isotropic spatial resolution allows for a visualization of the change of the transmural change of fiber orientation and high precision quantification of the myofiber helix angle. In an isoproterenol myocardial damage model, distinct change of myofiber orientation as represented by the helix angle in subendocardial regions was observed at different positions in the mouse heart (grey bars: control group. Red bars; isoproterenterol model). Reprinted under CC-BY license from (61). (**B**) DTI-based fiber-tracking in mouse aorta with 80 µm^3^ isotropic spatial resolution allows for visualization of the orientation of intimal vascular smooth muscle cells. (**C**) T2-weighted imaging of an excised porcine aorta with 0.3 mm slice thickness allows for an almost histologic visualization of vascular anatomy. (**D**) Dynamic MRI in the excised aorta following exposition to 30 mM TEMPOL as an T1-modifying contrast agent. The TEMPOL-induced change of the T1 relaxation times from outside and inside the vessel allows for the study of its kinetics over time. As a proof of principle, the aorta was exposed to 20 mM ascorbic acid (ASA) after 40 min. ASA also diffuses into the vascular wall with a similar time constant. As it reduces the free electron of TEMPOL, T1-contrast vanishes completely at 50 min. This type of measurement may allow for imaging of vascular or myocardial oxidative stress. (**E**) MRI of a 2 mm long cardiac organoid. MRI at 20 × 20 × 70 µm^3^ spatial resolution with the organoid exposed to buffer solution. The dark spot in the right image denotes an air bubble which also induces a slight susceptibility artefact. (**F**) Dynamic imaging of TEMPOL kinetics in the organoid of Fig. (**E**) after exposure of the organoid to TEMPOL dissolved within buffer. The red and the blue curves denote the temporal evolution of the MRI signal in two different regions, while the orange curve is a reference region in buffer. Please note that the buffer signal is higher than that the organoid because—other than in Figure 6E—buffer contained T1-shortening TEMPOL.

A variety of experimental techniques were also developed at the Bruker small-bore MRI system (Figure 6). As a consequence of the equal field strength of both MRI systems, techniques deliver similar results independent from the MRI system used. E.g., DTI was also feasible in mouse heart, even under remote conditions, i.e., after sacrificing the animals and fixing their hearts. These sacrificed hearts were shipped to our site and scanned successfully. Precise data on myofiber orientation can be obtained which help in the understanding of changes of microstructural orientation in disease or after intervention (Figure 6A). DTI in a mouse aorta also shows the intimal vascular smooth muscle fiber orientation (Figure 6B).

##### 3.3.4.2. Excised pig aorta

An almost histologic image resolution and visualization of distinct vascular structures was obtained within a scan time of 60 min (Figure 6C). The contrast mechanism of oxidative stress imaging using TEMPOL/ascorbic acid was well visualized. Since the vascular specimen was exposed to TEMPOL from both sides, it diffuses into the vascular wall is well visualized by T1-weighted imaging (Figure 6D). After 30 min, TEMPOL is also well visible in the tunica media. Subsequent exposition after 40 min to ascorbic acid results in a diffusion of that substance into the vascular wall as well, and TEMPOL is converted to diamagnetic hydroxyalamine. In consequence, T1 contrast vanishes (Figure 6D), indicating the presence of oxidative stress if the ascorbic acid model is considered as a surrogate to the presence of ROS.

##### 3.3.4.3. Cardiac organoids

Despite their small size of two millimeters, high-resolution MRI of cardiac organoids was feasible (Figure 6E). No mechanical contraction of the organoid was observed. As a test of imaging of passive substance diffusion into the organoid, semi-dynamic imaging during exposition of the organoid to TEMPOL was performed. TEMPOL diffusion into the organoid with a temporal resolution of 7 s was well feasible with the expected different kinetics at different distances to the surface of the organoid (Figure 6F).

### 3.4. Cardiac imaging in humans

**Figure 7:**
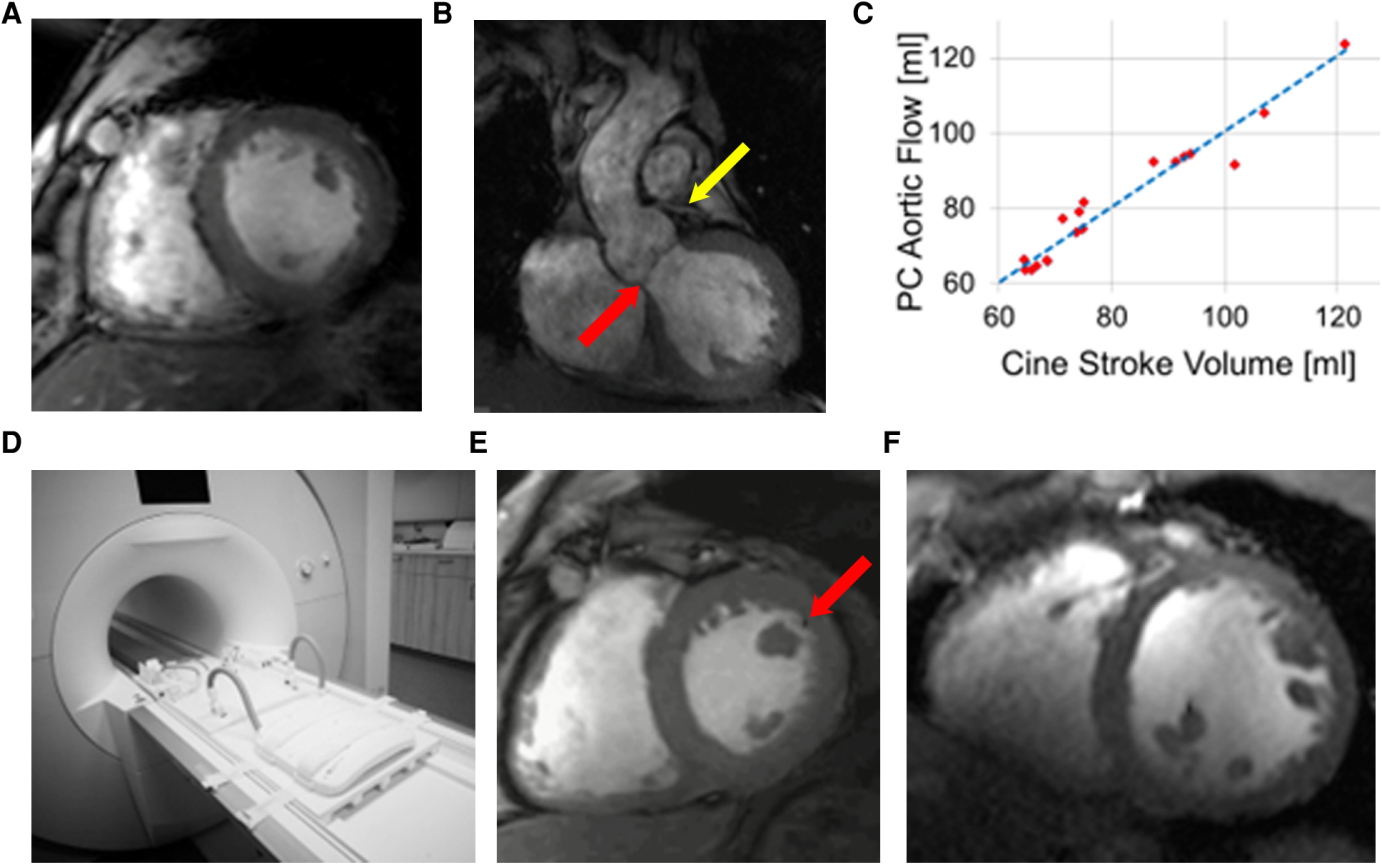
UHF-MRI in humans. (**A**) Short axis CINE imaging in a healthy volunteer shows signal inhomogeneities because of *B*_0_ and *B*_1_ inhomogeneities typical for human imaging at 7 T, but imaging of the left-ventricular outflow (**B**) tract nicely visualized the aortic valve (red arrow) and the left main coronary artery (yellow arrow). (**C**) Comparison of stroke volume as obtained from functional CINE imaging with phase-contrast flow measurement in the ascending aorta demonstrates excellent agreement and, thus, reliability of UHF-MRI-based quantitative analysis of this type of measurement. For these measurements in subfigures (**A–C**), the vendor-supplied 1Tx/16RX cardiac coil was used. Using a new 8 Tx/16Rx MRI coil (**D**) allows for better image quality in a patient (**E**) which was scanned during a routine check-up where ECG abnormalities were detected. In combination with subsequent clinical imaging [e.g., (**B**)], prior ischemic myocardial damage was diagnosed. Although the 3 T scan in this patient (**F**) is not of particularly good image quality, this image comparison demonstrates the potential of the image quality inherent in UHF-MRI. The black dot in the 7 T-image (red arrow) is an artifact from image reconstruction resulting from phase singularities of the “adaptive combine” coil combination technique.

Cardiac imaging in humans routinely delivers diagnostic image quality even with the commercially available 1Tx/16Rx RF coil without pTx (Figure 7 and Supplementary Video S3). Currently, measurement protocols for pulse sequence localization, shimming, as well as cardiac function measurement using myocardial CINE imaging and phase contrast flow measurements in the ascending aorta are established. On a larger data basis, the results in 84 examinations demonstrated good image quality with nondiagnostic image quality in only 3.4% of left-ventricular segments in 50 subsequent volunteers (23). Functional assessment for stroke volume agreed well between CINE and phase-contrast flow measurement (Figure 7C). Finally, Figures 7D–F demonstrates that the new commercial 8Tx/16Rx cardiac array can be efficiently applied in pTX compatibility mode to acquire cardiac images with better diagnostic quality.

### 3.5. Translational cardiovascular imaging across scales

**Figure 8:**
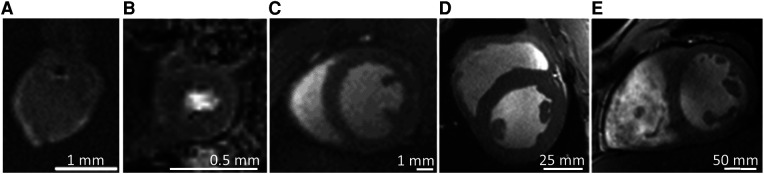
Summary of translational imaging concept. The described research infrastructure allows imaging for the full spectrum of samples relevant for modern translational research, from (**A**) cardiac organoid (30 × 30 × 300 µm^3^) with yellow marked regions of interest for subsequent data analysis in the central and peripheral region of the organoid, (**B**) Arrested zebrafish heart *ex-vivo* (25 × 25 × 50 µm^3^, c.f. Supplementary video S1), (**C**) CINE MRI in a mouse heart *in-vivo* (0.2 × 0.2 × 1.0 mm^3^, c.f. Supplementary video S2), (**D**) pig heart *in-vivo* (0.4 × 0.4 × 6 mm^3^), and (**E**) human heart *in-vivo* (0.6 × 0.6 × 6.0 mm^3^, c.f. Supplemental video S3). All imaging sections were positioned in a mid-ventricular short-axis view.

To demonstrate the potential of cardiovascular imaging at 7 T using the described infrastructure, Figure 8 demonstrates imaging of from 2 mm-sized cardiac organoids, zebrafish *ex-vivo* (c.f., Supplementary video S1), mouse heart *in-vivo* (c.f., Supplementary video S2), pig heart, and human heart (Supplementary video S3).

## 4. Discussion

We present here a research infrastructure concept for dual use of whole-body MRI systems in both, humans and pigs. We provide evidence that UHF-MRI can be used successfully in translational research in a variety of species of relevance in cardiovascular research: from millimeter-size cardiac organoids and zebrafish organs, to mice, pigs, and humans. Although UHF-MRI is not widely available yet, CINE and phase-contrast flow imaging provide image quality sufficient for functional assessment of the LV in humans. Moreover, if combined with dedicated size- and shape-adapted RF coils, UHF-MRI provides excellent image quality in serial imaging of porcine infarction *in-vivo* and in arrested or excised hearts. High-resolution pilot data from imaging in pigs have been presented, in particular from techniques well known in cardiac imaging such as first-pass perfusion and delayed enhancement MRI, susceptibility (T2*) weighted and diffusion tensor imaging. Moreover, proof-of-principle of ROS-like image contrast based on stable radicals such as TEMPOL has been presented.

### 4.1. General UHF-MRI and dual use concept

This appears to be the first report of a translational infrastructure dedicated for using UHF-MRI to obtain precision imaging in pigs. The results are promising, demonstrating that serial studies with animals, growing from small body size to almost adult size, can be performed with excellent image quality. We hypothesize that the better image quality and the higher spatial resolution in UHF-MRI may support high-precision functional assessment in pig models of cardiovascular disease. The higher precision may help in more detailed understanding of cardiovascular function, microstructure, and metabolism down to the cellular level. Moreover, the higher precision also may have an important aspect with regard to the 3R principle of animal welfare (64) in that statistical confidence may be obtained in smaller numbers of animals.

Our observed absence of Sars-Cov-2 infection in any of the pigs delivered during times of the first peak of the national pandemic situation, is promising from an animal experimentation point of view. However, it must be kept in mind that the vendor of the animals in our study was a vendor specialized in providing of laboratory animals and, thus, the risk of infection of an animal at the vendors' facility may be considered relatively low. However, our finding agrees with that of a recent review, which concludes that pigs are considered a low-risk species for Sars-Cov-2 spillover from pigs to man in xenotransplantation, but that monitoring of the risk appears essential because of continuous mutations of the virus (88).

### 4.2. Cardiac UHF-MRI in pigs

Large animal imaging is significantly more demanding than small animal or human imaging because of the complex interdisciplinary expertise needed for MRI-compatible instrumentation and experimentation (e.g., the use of non-metallic devices and implants), animal anesthesia, control of the animals physiology and pain medication during the MRI scan, and MRI expertise to perform the actual measurement with particular expertise and licensing for working with animals (79).

Animal experiments have an important role in animal research (89), although the role of imaging of animals in biomedical research (90) is not yet widely known in basic research despite the fact that it allows an important contribution in the context of the 3R-concept for animal experiments (82), namely to *refine* the data obtained from a single animal because with modern scientific and diagnostic imaging methods a deep understanding of tissue morphology, microstructure, function and metabolism can often be achieved in the individual animal. Moreover, compared to conventional experimentation in basic research, which often includes histological analysis, serial imaging of an animal (c.f., Figure 4) over an extended period of time is feasible and, thus, a *reduced* number of animals is needed. It goes without saying that MRI and histology often provide different information which may necessitate other study concepts if *in-vivo* imaging is used.

Pig models are an important part of both translational and imaging research., e.g., to assess post-infarction inflammation using fluorine-19 MRI (91), to understand functional and microstructural changes (92) or myocardial strain (93) following acute myocardial infarction, to understand pulmonary vasomotor control and ROS in exercising swine with multiple comorbidities (94), in resuscitation research (12, 95), to better understand imaging contrast such as the influence of myocardial blood flow on T2 (96), to develop medical devices, and as basis for computational modelling of the heart (7, 97–100).

Although the immune system and genetic code are more complex in large compared to small animals, a variety of large animal models exist for cardiac and cardiovascular research (101), e.g., for myocardial infarction (102, 103), and heart failure (104–106). Finally, the development and understanding of imaging methodology and technology is supported by the availability of large animal models for the above-mentioned reasons (9, 107), and because from a technical point of view, usually the same MRI systems and imaging techniques can be used in humans and large animals.

An interesting and probably essential observation in our study was the substantial correlation of both, localization and size of the $T_{2}^{*}$-contrasted regions with the regions of impaired myocardial perfusion as detected by DCE-MRI during days 3 and 60 after myocardial infarction. To the best of our knowledge, observations relating microcirculatory heterogeneity, bleeding during acute infarction and/or remodeling as monitored by $T_{2}^{*}$ contrast have not been reported so far and need to be investigated systematically in a larger study in the future. Moon and coworkers have already made some attempts towards that direction using ex-vivo imaging, but their study was limited to the early phase (2–4 h) of myocardial infarction (108).

### 4.3. Experimental UHF-MRI in pig hearts

MRI of arrested or excised pig hearts is less established in the research community (109) but is an excellent instrument to understand the underlying concepts of image contrast, e.g., in diffusion tensor imaging of the heart (98, 106) or blood vessels (110). It offers better access to the organ and measurements free of motion effects (49), although potential variations of quantitative tissue parameters may need consideration (56). Our study focused on ex-vivo models of pig hearts. The imaging methodology is not limited to pig hearts, however, and concepts may well be used in other well established ex-vivo canine (111), sheep (112), dog (113), or even human hearts (114).

Imaging *ex-vivo* but *in-situ*, i.e., with the heart still at its anatomic position, provided images free from motion-induced artifacts (Figure 3E). Under these conditions, is not limited by restricted breathholds and, thus, provides ground-truth data to better understand *in-vivo* imaging and image artifacts related to it. It is interesting to note that depending on the perfusate (e.g., high potassium or lithium perfusate), the heart can be fixed in slack or contracted state, respectively (98).

7 T MRI of excised pig hearts provided very high spatial resolution, lack of motion-induced artifacts and promising image contrast, both in susceptibility-weighted and in morphological imaging of infarcted myocardium (c.f, Figure 5). The clarification of the exact contrast mechanisms in both cases is beyond the scope of the current study and needs to be studied in further work. Image contrast in susceptibility-weighted imaging of healthy hearts may be a consequence of transmural variation of the myofiber helix angle or variations of T2* following tissue fixation (56). Due to immersion fixation, endocardial and epicardial tissue is exposed to the fixation process first, and thus that the degree of fixation and the amount of formalin present in these areas may vary although immersion was performed for at least seven days. Beating pig heart models are important for research on aspects where better access to the heart and direct control of its physiology are relevant factors, e.g., to better understand 4D flow patterns (115) or cardiac metabolism (98, 109, 115, 116). Here, the large bore and the excellent image quality obtainable at a whole-body UHF-MRI system may be highly advantageous to obtain even better physiological data.

### 4.4. UHF-MRI in humans

We present cardiac UHF-MRI data in humans. As expected, image quality is highly dependent of the coil used (c.f. Figure 7). Using a new 8 Tx/16Rx channel cardiac coil with centric symmetry, which now is available commercially, resulted in excellent image quality although even with a simpler 1Tx/16 Rx cardiac coil diagnostic image qualtiy was achieved (23). Thus, functional assessment using UHF-MRI at 7 T can be considered ready for patient use although large clinical studies are still missing. Further optimization of RF coils for clinical usage using dedicated customer *B*_1_-vectors may lead to further improvement of image quality in specific patient cohorts.

Although the potential of cardiac UHF-MRI at 7 T *in humans* has been demonstrated more than a decade ago (20, 117, 118), cardiac UHF-MRI still is not widely used in both research and clinical assessment of patients. Technical and methodological challenges still make the exam demanding and thus, it may be difficult to provide consistent image quality in all subjects across the whole heart. Moreover, safety procedures are not widely established, and no globally applicable guidelines exist. Alternatively, such as the MRI system used here a new generation of UHF-MRI systems has become available which are more suited to the high demands of a cardiovascular examination, and which also provide better image quality (23, 49, 119) although both vendors currently do not provide FDA or CE clearance for routine cardiac patient use. For human use, only MRI systems with a field strength of 3 T and less have been approved for cardiac applications by the FDA or the European Commission (CE). It can be hypothesized that cardiac UHF-MRI will be used more widely as soon as researchers and system vendors have provided consistent methodology regarding safety, whole heart image quality and contrast. Further key developments will be optimal RF coils, more stable techniques for synchronization of image acquisition with the heartbeat, and the full availability of the wide variety of MRI exams available at lower field strengths, in particular late-gadolinium enhancement and semiquantitative or quantitative perfusion imaging, or microstructural imaging based on the diffusion tensor or susceptibility weighting.

Recently, a new 5 T prototype whole-body MRI system equipped with a strong rapidly switching gradient system (120 mT/m, 200 mT/m/ms peak gradient strength and slew rate, respectively), 8 Tx volume coil and 24 + 48 channel Rx coil, and five 2nd and 3rd order shim coils has been presented. Initial results were promising in that CINE imaging in 17 healthy volunteers proved to be as good or better than 3 T (120). More data are needed to decide if 5 T is a better option than 7 T for clinical or translational research based imaging.

Although clinically not widely used, assessment of cardiac metabolism or ion hemostasis based on MR spectroscopy using ^31^P (121–123) or imaging of ^23^Na (124, 125), respectively, profit significantly from the high field strength and may make these applications better accessible to clinical research or patient diagnostics. It may be easier to implement those techniques at 7 T than normal clinical imaging because of the lower Larmor frequency of those heteronuclei.

The increase of SNR in clinical UHF-MRI when compared to normal clinical field strength may appear somewhat limited in terms of the underlying physics (approximately by a factor of 2 … 4) and may sound marginal and far from orders of magnitudes improvement. Even if the SNR improvement would only be a factor of two, this would mean that, e.g., the in-plane spatial resolution might be reduced (i.e., improved) to 71% of that at 3 T without losing SNR. Figure 4 demonstrates that this significantly improves the delineation of small structures such as the coronary arteries. The higher spatial resolution and higher SNR with subsequently more stable quantitative assessment of tissue parameters may have the potential to provide high-precision functional and structural assessment of patients. This precision imaging approach may become important in early diagnosis making to prevent severe stages of disease, e.g., in heart failure. Moreover, precision imaging may allow earlier assessment of therapy response because of the higher sensitivity to treatment-induced changes of cardiac function and structure. Thus, this kind of precision imaging may become an important monitoring and decision-making tool in personalized medicine approaches (126), such as cardiovascular pharmacotherapy (127), including assessment of sex related differences (128), or treatment of cardiomyopathies (129–131).

It may be speculated that cardiac MRI applications which currently are not part of the clinical repertoire, may cross a barrier because in the future, these applications may make a significant clinical impact considering the higher spatial resolution available at 7 T, e.g., coronary angiography (21), phase-contrast MRI of intracoronary blood flow (132), or MRI-based assessment of coronary flow reserve. Other applications may benefit from the higher SNR directly, e.g., quantitative myocardial perfusion MRI (133–136), diffusion tensor imaging (137–139), magnetic resonance spectroscopy (140) or MRI (141) of dilated cardiomyopathy or hypertrophic cardiomyopathy (142, 143), or cardiac amyloidosis (144). However, it cannot be excluded that new contrast mechanisms or physiological mechanisms will be detected in the future. Similar experience has been made with the detection of physiological noise in the early days of 3 T MRI in the brain (145).

Imaging of magnetic susceptibility may gain more interest also in cardiac UHF-MRI because its inherently high sensitivity to tissue induced variations of the magnetic susceptibility, although it can be challenging to discriminate tissue-based variations of the magnetic susceptibility from signal variations induced by macroscopic inhomogeneities of *B*_0_ in regions with interfaces between tissues with different magnetic susceptibilities e.g., in the posterior heart with its nearby heart/lung interface (20). Susceptibility-weighted imaging depends on variations of the microscopic distribution of magnetic susceptibilities, e.g., if myofibers are arranged different in disease such as HCM (143). It may be of interest to discriminate between cardiac amyloidosis and HCM (146), to assess iron overload, myocardial oxygenation (59, 120, 147), or to discriminate between bleeding and remodeling after infarction (148) (c.f., Figure 3), although the discrimination between confounding and pathology-relevant factors may be difficult.

### 4.5. Translational concept—from mouse to man

With the described infrastructure concept, cardiac imaging was feasible across a large spectrum of objects and species important for translational cardiac research (c.f., Figure 8). Although the full capacity of this wide spectrum of applications has not yet been used, it has become clear that two parts of our concept are helpful to streamline our research: (i) the same field strength of the MRI systems, which often allows to transfer MRI findings (e.g., measurement parameters, tissue parameters like relaxation times or contrast agent relativities). (ii) The dual use concept for imaging of both, pigs and humans. The simple transfer and exchange of measurement techniques developed for one species to the other significantly reduced the amount of imaging research needed, and competences of researchers are found easier than if another MRI system would be available for the measurements. Moreover, since animals often are smaller than humans, in particular early after their delivery to our institution, 7 T-related issues such as dielectric resonances often are less pronounced compared to the adult human situation. This reduces the complexity of cardiac UHF-MRI in some conditions. Moreover, in the current situation with not yet completely established safety and SAR-reduction procedures, pig imaging is a serious competitor to human studies in pathophysiological studies. Moreover, imaging of arrested or excised hearts, and imaging of cardiac tissue samples, allows for spatial resolution, microstructural and functional information to be obtained with a quality not feasible currently under *in-vivo* conditions.

Key to these imaging technologies are optimized RF coils, adequate organizational concepts in particular with regard to dual use of clinical MRI systems in human and animal work, and well-trained personnel.

### 4.6. Limitations of the study

This study is a description of important components of a scientific infrastructure dedicated to translational cardiovascular research. We demonstrate the feasibility of mainly large animal imaging at UHF-MRI, since to our knowledge no systematic literature on that exists. Moreover, we demonstrate pilot measurements both in large animals and measurements for translational cardiovascular research becoming feasible in such an infrastructure. The thorough analysis of the animal models, and analysis and interpretation of the data obtained from MRI and in animals, however, are beyond the scope of this manuscript and will be presented later (85).

A major limitation of this study cardiac UHF-MRI is certainly that cardiac 7 T MRI still is in development and large patient studies demonstrating clinical feasibility are still missing. However, we think that the technique is already useful, in particular in the translational research arena where large animal imaging is needed. Here, the higher field strength already now is advantageous compared to clinical 1.5 T and 3 T MRI, or recent 5 T scanners (149).

## 5. Conclusion

In conclusion, translational UHF-MRI is feasible and large animal experiments can be conducted with excellent data quality. Using an UHF-MRI system in both large animal and human imaging reduces costs because similar techniques and the same personnel can be used in both. Moreover, because of the shared application, quicker development of UHF-MRI technologies and their applications in humans is feasible. UHF-MRI may provide data for translational research relevant for a wide range of cardiovascular research questions. Wider patient use for clinical cardiac decision making is on the horizon, given currently available UHF-MRI systems.

## Data Availability

The datasets presented in this article are not readily available because the current ethics approvals do not allow for sharing of human data. If a request would be received, it would be forwarded to the local ethics committee. Data from animal experiments are available on request. Requests to access the datasets should be directed to Schreiber_L@ukw.de.
