# Supplementary material for: Ultra-high field cardiac MRI in large animals and humans for translational cardiovascular research
Source: Front Cardiovasc Med. 2023 May 15;10:1068390. doi: 10.3389/fcvm.2023.1068390 (PMC10225557; doi:10.3389/fcvm.2023.1068390)
Supplement: Supplementary file 1 [file Datasheet1.docx]

Supplementary Material

# Mouse heart and zebrafish fixation setup

For MR measurements, we used a 5 ml syringe. In a first step the syringe is filled with Fomblin™ (Solvay Specialty Polymers, Italy) after which the outlet of the syringe is closed using a stopper. In the next step, the syringe is held with outlet turned towards the floor and the syringe plunger is removed. After rinsing the fixed zebrafish using saline solution and drying the fish using gauze, the sample is placed in the syringe via the plunger access and fixed using a small piece of Fomblin soaked gauze. Afterwards the syringe plunger is slightly inserted again. The syringe is then turned with the outlet upwards, and the stopper is removed. The plunger is now moved into the syringe, removing access air, after which the stopper is reapplied and the sample ready for measurement. The syringe is placed on a 3D printed ABS sledge designed inhouse. This sledge allows optimal placement with respect to the cryogenic coil, which was used for image acquisition. The images below show the prepared sample on the inhouse built sledge (grey), which is mounted on the vendor sledge of the cryogenic coil. Image B shows a top view of syringe and specimen positioning, as well as a close-up of the specimen inside the syringe. The same setup has been successfully applied to fixed mouse hearts using a 2 ml syringe.

A


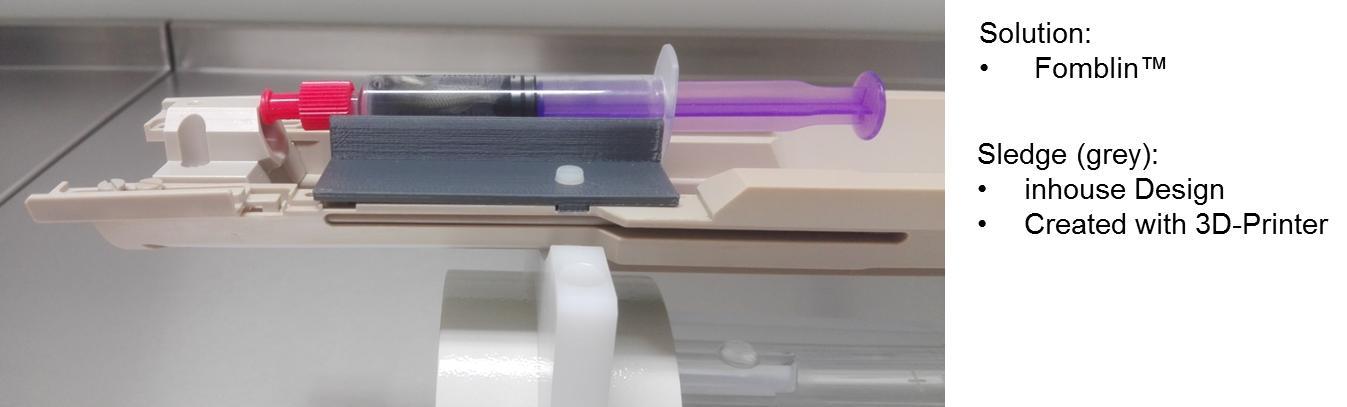


B


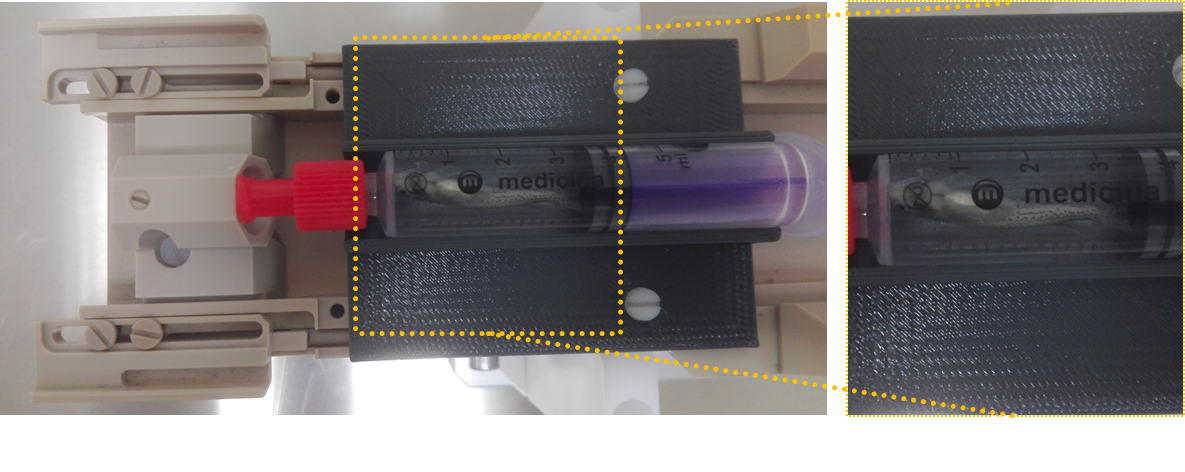


Measurement parameters for zebrafish imaging using a 3D gradient echo pulse sequence with 25x25x25ym^3^ isotropic spatial resolution were TE/TR: 13/90, matrix size: 320x320, FOV 8x8mm^2^, slab thickness: 4mm, 160 slices, averages: 38. Total scan time was 54 hours.

# MRI of TEMPOL-exposed cardiac organoids

Foreskin human fibroblast derived induced pluripotent stem cells (FS hiPSC, clone 2) were generated from normal human dermal fibroblasts (NHDF; Promocell, Heidelberg, Germany) by reprogramming using hSTEMCCA‐lentiviral construct^1^ with in-house developed method. 2.5x10^5^ to 2.5x10^6^/well FS iPS were seeded in 0.5ml/well of StemMACS iPS-Brew (Miltenyi, 130-104-368) for 24h for aggregate formation. After aggregate formation, the cells were seeded on a 24 well plate pre‐coated with 1% agarose (Sigma–Aldrich, St Louis, MO, USA) in cardiac basal medium (CBM) containing enriched RPMI1640 (Gibco®, Waltham, MA, USA)^2^. The maintenance of the cardiac organoids consisted in further cultivation in 1ml CBM and medium exchange every 3-4 days.

TEMPOL (4-Hydroxy-2,2,6,6-tetramethylpiperidine1-oxyl´) was dissolved at 1M in NaCl stock solution and stored at 4ºC. Subsequent dissolutions with cell culture medium were prepared prior to each measurement.

Native, 15mmol TEMPOL-exposed cardiac organoids with a diameter of 2mm were scanned in 100µl CBM in a 1ml sealed syringe for no longer then 50min in order to avoid (1) oxygen, (2) glucose tension and (3) temperature shock. For the high-resolution scan, RARE with two subsequent refocusing pulses per repetition time was used as SE sequence with following parameters: TR/TE=323/10ms, TA=45min, FA=90°, matrix size=256x160, field-of-view=6x8mm, slice thickness=0.15mm. For kinetic scans, a GRE pulse sequence with the following parameters was used: TR/TE=90/3.54ms, TA=302sec, FA=90°, matrix size=160x140, field-of-view=7x7mm, slice thickness=0.3mm.

**References:**1. Sommers, A. et al. Generation of Transgene-free Lung Disease-Specific Human Induced Pluripotent Stem Cells Using a Single Excisable Lentiviral Stem Cell Cassette. [Stem Cells.](https://www.ncbi.nlm.nih.gov/pubmed/20715179) 2010 Oct;28(10):1728-40. doi: 10.1002/stem.495.

2. Kadari, A. et al.: Robust Generation of Cardiomyocytes from Human iPS Cells Requires Precise Modulation of BMP and WNT Signaling. [Stem Cell Rev Rep.](https://www.ncbi.nlm.nih.gov/pubmed/25392050) 2015 Aug;11(4):560-9. doi: 10.1007/s12015-014-9564-6.

# 3 Supplemental Video 1 – Zebrafish MRI

T1-weighted axial sections through a zebrafish with very high spatial resolution of 25 x 25 x 50 µm^3^ obtained within 16 hours. Excellent anatomical detail of the heart and whole body is observed.

# 4 Supplemental Video 2 – In-vivo MRI of the mouse heart.

Bright-blood CINE-MRI of a mouse heart obtained at 0.2 x 0.2 x 1 mm^3^ spatial resolution. For this prospectively cardiac and breath-gated acquisition with TR/TE = 10 ms/ 1.4 ms a home-built double-tuned ^23^Na/^1^H surface coil was used.

# Supplemental Video 3 – In-vivo MRI of the human heart.

High resolution CINE imaging in a human volunteer. Compared to clinical field strengths where 6 mm or thicker slice thickness is usually selected, thin slices of 4mm thickness become feasible. In consequence, more distinct visualization of papillary muscle in both ventricles and higher overall image sharpness is observed. While higher resolution in cardiac function may offer new insights into pathology and increase the accuracy in derived metrics of cardiac function, the smaller voxel volumes are also beneficial to reduce B_0_-related susceptibility effects, particularly at lung tissue interfaces. Images were acquired using a spoiled gradient echo sequence with in-slice flow compensation. The sequence was retrospectively gated and set to generate 30 cardiac phases within 10.2s breath holds. Measurement parameters were TE/TR: 2.9/44.1 ms, bandwidth: 915 Hz/Px, slice thickness 4 mm, FOV: 377x400 mm^2^ and an (interpolated) in-plane resolution of 0.7x0.7 mm^2^_._
